# Update on adherence to guidelines for time to initiation of postoperative radiation for head and neck squamous cell carcinoma

**DOI:** 10.1002/hed.27380

**Published:** 2023-04-27

**Authors:** F. Jeffrey Lorenz, Sean S. Mahase, Joseph Miccio, Tonya S. King, Sandeep Pradhan, Neerav Goyal

**Affiliations:** 1 College of Medicine, The Pennsylvania State University, Hershey, Pennsylvania, USA; 2 Department of Radiation Oncology, Penn State Hershey Medical Center, Hershey, Pennsylvania, USA; 3 Department of Public Health Sciences, Penn State Hershey Medical Center, Hershey, Pennsylvania, USA; 4 Department of Otolaryngology – Head and Neck Surgery, Penn State Hershey Medical Center, Hershey, Pennsylvania, USA

**Keywords:** head and neck cancer, head and neck squamous cell carcinoma, postoperative radiation, quality care, treatment delay

## Abstract

**Background:**

A prior study reported that over half of patients with HNSCC initiated PORT after 6 weeks from surgery during 2006–2014. In 2022, the CoC released a quality metric for patients to initiate PORT within 6 weeks. This study provides an update on time to PORT in recent years.

**Methods:**

The NCDB and TriNetX Research Network were queried to identify patients with HNSCC who received PORT during 2015–2019 and 2015–2021, respectively. Treatment delay was defined as initiating PORT beyond 6 weeks after surgery.

**Results:**

In NCDB, PORT was delayed for 62% of patients. Predictors of delay included age >50, female sex, black race, nonprivate insurance/uninsured status, lower education, oral cavity site, negative surgical margins, increased postoperative length of stay, unplanned hospital readmissions, IMRT radiation modality, treatment at an academic hospital or in the Northeast, and surgery and radiation at different facilities. In TriNetX, 64% experienced treatment delay. Additional associations with prolonged time to treatment included never married/divorced/widowed marital status, major surgery (neck dissection/free flaps/laryngectomy), and gastrostomy/tracheostomy dependence.

**Conclusions:**

There continue to be challenges to timely initiation of PORT.

## 1 | INTRODUCTION

Timely, guideline-adherent treatment is essential to delivering quality cancer care.^1^ According to the National Comprehensive Cancer Network (NCCN), patients with locoregionally advanced head and neck squamous cell carcinoma (HNSCC) requiring postoperative radiation (PORT) should initiate therapy within 6 weeks of surgery.^2^ A National Cancer Database (NCDB) analysis spanning 2006 through 2014 reported that greater than half of patients with HNSCC requiring PORT initiated treatment after 6 weeks from surgery.^3^ Several subsequent publications attempt to further elucidate reasons for treatment delay and measures to implement targeted quality improvement interventions.^4–6^

In January of 2022, the American College of Surgeons (ACS) Commission on Cancer (CoC) released a national quality metric for patients with HNSCC to initiate PORT within 6 weeks.^7^ Given that 8 years passed since the last large-scale analysis of timeliness of care, this study provides an update on adherence to guidelines for time to initiation of PORT for patients with HNSCC. Additionally, we sought to define the current state of the problem, propose additional factors associated with delay, and need for further interventions.

## 2 | MATERIALS AND METHODS

The study data was obtained from the NCDB. The NCDB includes patient information from more than 1500 CoC accredited institutions across the United States.^8^ The study cohort, outcome measures, and study variables were based on methods previously described by Graboyes et al.,^3^ except for the time period of database review. Graboyes et al. analyzed 2006 through 2014; therefore, we reviewed 2015 through 2019. The cohort was composed of patients with HNSCC who initiated PORT, with treatment delay defined as beginning radiation beyond 6 weeks after the date of surgical intervention. Demographic characteristics collected included age, sex, race, ethnicity, insurance status, area of residence, educational attainment quartile, median household income, and distance to hospital. Educational attainment was determined by comparing patient zip codes at the time of diagnosis to American Community Survey data and is based on the percentage of adults in a given zip code who did not graduate high school. Clinical and surgical covariates included cancer site, American Joint Committee on Cancer (AJCC) clinical and pathological stages, surgical margin status, postoperative length of stay, 30-day hospital readmissions, radiation modality, concurrent chemoradiation, and Charlson–Deyo Comorbidity score. Patients diagnosed before 2018 were staged according to the AJCC 7th edition guidelines, and after 2018 using the 8th edition. Hospital characteristics analyzed included treatment facility type, number of facilities involved in treatment, whether surgery and radiation were conducted at the same facility, and region of care. Treatment facility type is based on the annual volume of newly diagnosed cancer cases and structure of facilities: community cancer program (100–500 annual diagnoses), comprehensive community cancer program (>500 annual diagnoses), academic/research program (>500 annual diagnoses plus resident trainees), and integrated network cancer program (organization owning a group of facilities designed to offer comprehensive and integrative cancer care services).

An additional analysis was conducted using the TriNetX Research Network. TriNetX is a global federated health research database that provided access to electronic medical records (diagnoses, procedures, medications, laboratory values) from health care organizations (HCOs) in the United States.^9^ The database was queried to identify patients with head and neck cancer diagnosed during 2015 through 2021 who initiated PORT. Similar exclusion criteria were applied as the NCDB analysis, that is, those who underwent surgery beyond 6 months after initial diagnosis or radiation beyond 6 months postoperatively, and those who underwent induction chemotherapy or received stereotactic radiation or brachytherapy. The diagnosis and procedure codes utilized to execute this search criteria are listed in Data S1, Supporting Information. Those who met inclusion and exclusion criteria were stratified according to whether they received PORT within or beyond 6 weeks of surgery. Demographic (age, sex, race, ethnicity, region, marital status), clinical (tumor site, concurrent chemoradiation, and presence of tracheostomy or gastrostomy tube), and surgical variables (neck dissection, free flaps, laryngectomy) were compared between groups.

## 3 | STATISTICAL ANALYSIS

For the NCDB analysis, bivariate associations were first assessed using chi-square tests to compare between those with initiation of PORT less than or equal to 6 weeks versus beyond 6 weeks. The factors with significant bivariate associations were then considered for the multivariable logistic regression modeling process. All factors were included in the full model, which was subsequently reduced using a manual backwards selection procedure to only include those factors which remained significant at the 0.05 level. Analysis was conducted utilizing SAS statistical software version 9.4 (SAS Institute Inc., Cary, NC). For the TriNetX subanalysis, odds ratios and 95% confidence intervals were calculated to determine the odds of receiving substandard care for those who belonged to a particular demographic or had certain clinical or surgical variables coded in their medical record. Statistical analyses were performed within the TriNetX platform, which is based on R, JAVA, and Python.

## 4 | ETHICAL APPROVAL

Both TriNetX and the NCDB only use aggregated counts of de-identified information, with no protected health information or personal data made available. Therefore, this study was exempted by the Penn State Institutional Review Board review (STUDY00018629 for TriNetX and STUDY00018678 for NCDB).

## 5 | RESULTS

### 5.1 | NCDB analysis

In the NCDB, 47 331 patients were identified as potentially eligible for analysis. Excluded were 4770 patients who underwent brachytherapy, stereotactic radiosurgery, or had an unknown radiation modality, 570 patients who received palliative treatment, and 249 patients who underwent neoadjuvant therapy. There were an additional 1244 patients who underwent surgery beyond 6 months of diagnosis and 334 who began radiation beyond 6 months of surgery who were also excluded. The final cohort for analysis included 40 164 patients, with demographics described in Table 1.

In this database, 62.0% (*n* = 24 901) of patients began PORT beyond 42 days (6 weeks) of surgery. By 8 and 10 weeks, 31.7% and 15.9% of patients had not yet initiated PORT, respectively, with the complete breakdown presented in Table 2. Bivariate comparisons of demographics, clinical and surgical characteristics, and hospital characteristics of those treated with PORT within or beyond 6 weeks are presented in Tables 1, 3, and 4, respectively (as well as Tables S1–S3, which are the same tables presented with percentages out of all patients).

**FIGURE 1:**
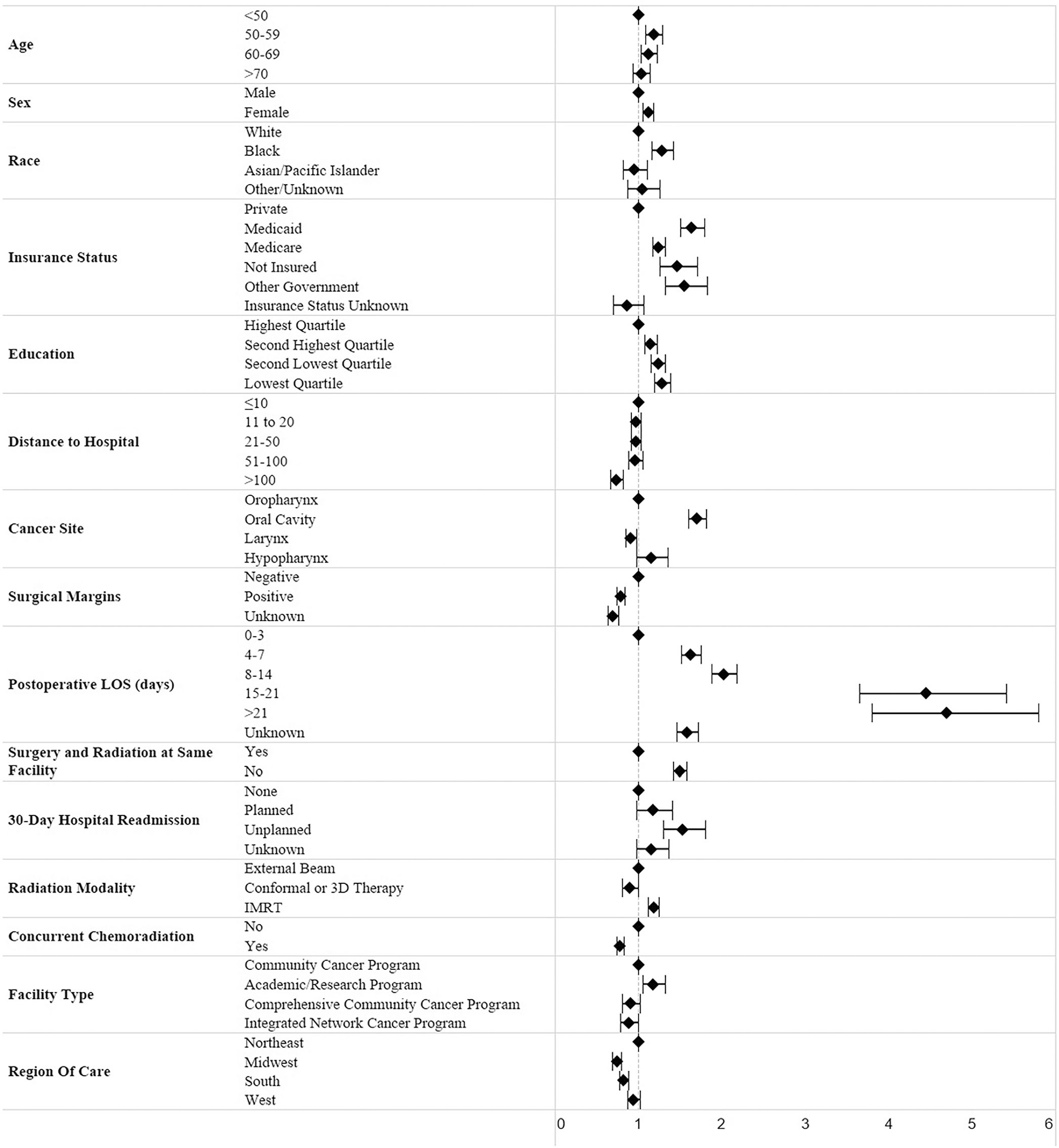
Predictors of PORT delay on multivariable analysis

On multivariable analysis, significant demographic predictors of timeliness of PORT included (OR >1 indicative of delay) age 50–59 years (OR, 95% CI) (1.18, 1.09–1.29) and 60–69 years (1.12, 1.03–1.22) compared to <50 years, female sex (1.12, 1.06–1.19) compared to male, black race (1.28, 1.16–1.41) versus white, Medicaid (1.64, 1.50–1.79), Medicare (1.24, 1.17–1.32), other government insurance (1.56, 1.33–1.82), and uninsured status (1.46, 1.25–1.70) compared to private insurance, belonging to the lowest quartile of educational attainment (1.28, 1.19–1.39) compared to the highest, and distance to the treatment center >100 miles (0.74, 0.67–0.82) compared to ≤10 miles. Clinical and surgical factors that remained significant included oral cavity cancer site (1.70, 1.60–1.81) compared to oropharynx, positive surgical margin status (0.79, 0.74–0.83) compared to negative margins, postoperative length of stay 4–7 days (1.63, 1.52–1.75), 8–14 days (2.02, 1.88–2.18), 15–21 days (4.44, 3.65–5.41), and >21 days (4.69, 3.80–5.80) compared to 0–3 days, unplanned hospital readmission (1.53, 1.30–1.80) compared to no readmission, IMRT radiation modality (1.18, 1.12–1.25) compared to external beam, and concurrent chemoradiation (0.78, 0.74–0.83) versus not receiving it. Significant hospital characteristics included treatment at an academic center (1.18, 1.05–1.32) compared to community cancer program, surgery and radiation at the different facilities (1.50, 1.42–1.57) compared to same facility, and Midwest (0.74, 0.69–0.80) and South (0.82, 0.77–0.88) regions of care compared to Northeast. The specific subcategories associated with delay are presented with their respective effect sizes in Figure 1. Factors that were not associated with delay on multivariable analysis included ethnicity, urban/rural area of residence, household income, Charlson–Deyo comorbidity score, clinical and pathological stage, and number of CoC facilities involved in treatment.

**FIGURE 2:**
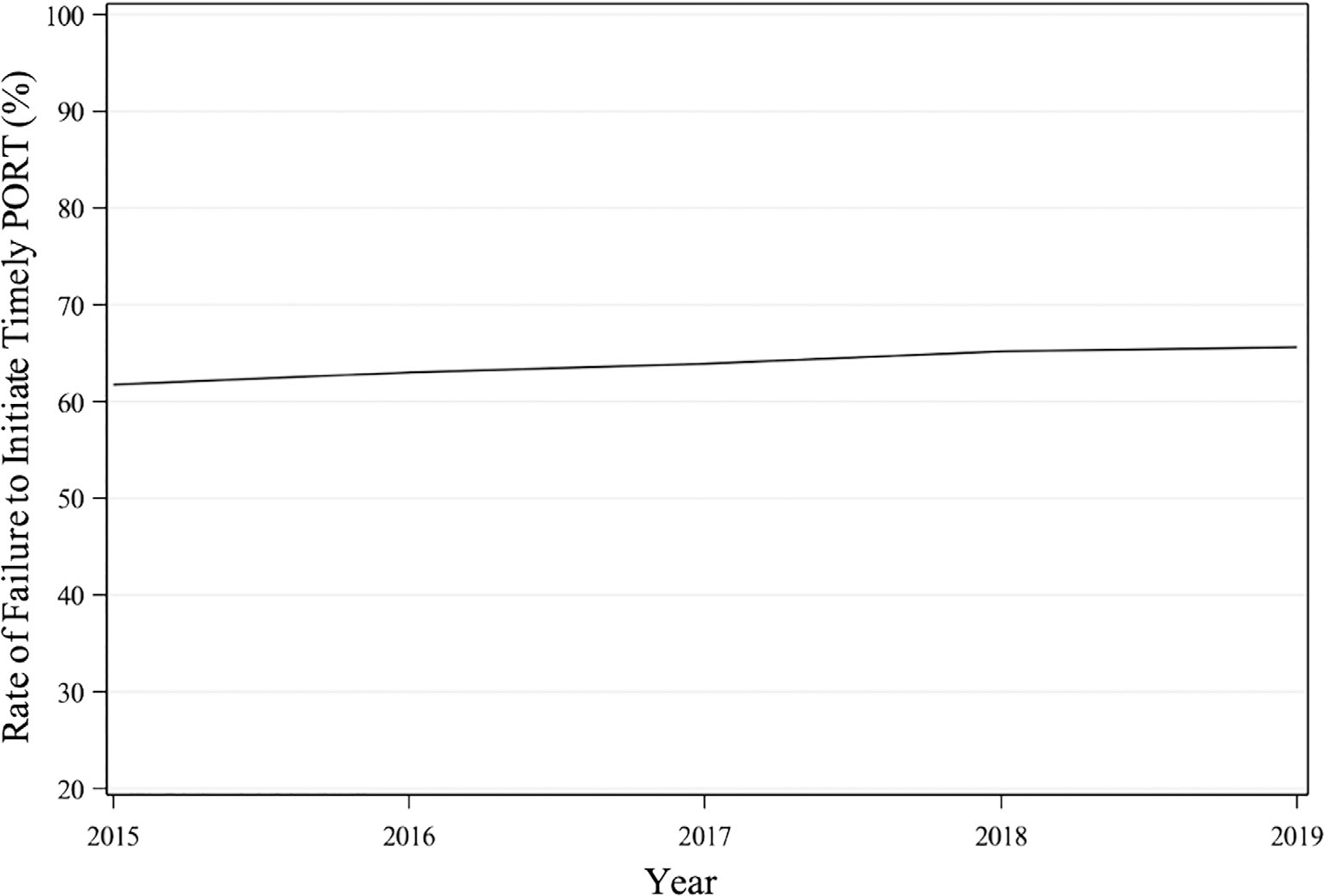
Temporal trends in the failure to initiate PORT within 6 weeks of surgery according to year of diagnosis

Analysis of temporal trends indicated a gradual increase in the annual percentage of patients who failed to initiate PORT within 6 weeks from 2015 to 2019 (Figure 2).

Analysis of risk factors for prolonged LOS and readmissions is presented in Tables 5 and 6 (and Tables S4 and S5). Among patients with oral cavity or hypopharynx tumor sites, 43.6% and 46.2% had postoperative LOS at least 8 days, compared to just 8.9% and 28.8% for oropharynx and larynx, respectively. Additionally, 3.2% and 4.2% of patients with oral cavity and hypopharynx tumor sites had unplanned hospital readmissions, compared to 2.1% and 2.3% of patients with oropharynx and larynx tumor sites, respectively. Additional patients more likely to experience a hospital stay ≥8 days included those of black race (42.9% vs. 25.9% of white patients), Hispanic ethnicity (35.5% vs. 27.5% of non-Hispanic patients), who were uninsured or had Medicaid (40.5% and 43.8%, respectively, compared to 21.4% for private insurance), who belonged to the lowest educational quartile (34.8% vs. 23.0% for highest educational quartile), who earned an income <$40227 (35% vs. 24.1% for income ≥$63333), who lived ≥100 miles from the hospital (34.4% vs. 25.9% for ≤10 miles), with a Charleson–Deyo comorbidity score of ≥2 (37.0% vs. 25.3% for score of 0), with clinical and pathological disease stage of 4 (34.1% and 32.8%, respectively, compared to 8.5% and 11.1% for clinical and pathological stage of 1), who had negative surgical margins (33.4% vs. 19.6% for positive margins), who had unplanned readmissions (43.7% vs. 27.4% for no readmission), and who underwent external beam or IMRT (26.1% and 29.8%, respectively vs. 14.3% for conformal or 3D therapy). Additional patients more likely to experience an unplanned hospital readmission included those of black race (3.8% vs. 2.6% for white), with Medicaid insurance (3.3% vs. 2.4% for private), who lived in rural settings (3.4% vs. 2.5% for urban), belonged to the lowest educational quartile (3.1% vs. 2.6% for highest quartile), who made <$40227 (3.3% vs. 2.6% for income ≥$63333), who lived >100 miles from the treatment center (3.2% vs. 2.6% for ≤10 miles), with a Charleson–Deyo comorbidity score of ≥2 (4.0% vs. 2.4% for a score of 0), with clinical or pathological disease stage of 4 (2.9% and 3.0%, respectively, vs. 1.2% and 1.5% for disease stage of 1), with negative surgical margins (3.0% vs. 2.5% for positive margins), with increased postoperative LOS (5.9% for LOS > 21 days vs. 1.6% for LOS 0–3 days), and who underwent IMRT (2.9% vs. 2.0% conformal or 3D therapy).

### 5.2 | TriNetX analysis

In TriNetX, there were 3651 patients with head and neck cancer who were treated with surgery and PORT during 2015 through 2021. Of this cohort, 486 patients were excluded for the following reasons: received stereotactic radiation or brachytherapy (*n* = 14), received induction chemotherapy (*n* = 35), and underwent surgery beyond 6 months after initial diagnosis (*n* = 115) or PORT beyond 6 months of surgery (*n* = 322). This left 3165 patients who met criteria for analysis. Demographics are described in Tables 7 and S6.

Similar to NCDB, 63.9% (*n* = 2023) of patients in TriNetX initiated PORT greater than 6 weeks after surgery. By 8 and 10 weeks, 35.5% and 19.0%, of patients still had not initiated PORT, respectively (Table 2). Additional unique predictors of delay (OR, 95% CI) included never married (1.51, 1.19–1.92), divorced (1.41, 1.02–1.92), or widowed (1.47, 1.01–2.13) marital status compared to married, major surgical procedures including neck dissection (1.55, 1.34–1.79), laryngectomy (2.01, 1.54–2.64), and free osteocutaneous (1.68, 1.30–2.18), myocutaneous (2.59, 1.72–3.91), or fascial flaps (2.27, 1.32–2.88) compared to those who did not undergo these operations, and gastrostomy (1.30, 1.11–1.51) or tracheostomy (1.67, 1.41–1.96) dependence compared to those without (Tables 7 and 8 and S6 and S7).

## 6 | DISCUSSION

Substantial strides in cancer care resulted from the advent of novel treatments, adoption of the multidisciplinary approach to patient-centered care, and implementation of evidence-based practice guidelines. However, variability in adherence to guidelines results in suboptimal outcomes across various malignancies.^10^ In the setting of HNSCC, initiating PORT within 6 weeks of surgical resection is associated with improved locoregional control and overall survival.^11–17^ Graboyes et al. reported that time to PORT deviated from this guideline for approximately half of this patient cohort during 2006–2014.^3^ Our updated analysis demonstrated a persistence of this trend, with nearly two-thirds of patients initiating PORT beyond the 6-week timepoint.

In our study, a variety of demographic, clinical, surgical, and hospital factors predicted treatment nonadherence, with many variables sharing commonality with the prior NCDB analysis.^3^ Persistent associations with treatment delay included black race, public insurance, lower educational attainment, oral cavity tumor site, increased postoperative length of stay or unplanned readmissions, treatment at an academic center, and fragmented surgery and PORT at different locations. The parallels of our findings 5 years after the initial report underscores inadequate interventions to rectify this disparity despite expanding evidence dedicated to understanding patient and system-level barriers to timely PORT.^3–6^ Additionally, temporal trends are suggestive of worsening adherence over time. Variables with the strongest associations with PORT delay included increased length of hospitalization, unplanned readmission, oral cavity cancer site, and insurance status. When designing interventions, targeting these areas may provide the greatest opportunity for improvement.

There are reports of a few isolated but promising quality improvement endeavors resulting in reduced PORT delay. One institution reported that the percentage of free flap patients receiving timely PORT increased from 10.5% to 50% due to three interventions: standardized care-pathway order sets, prompt referrals to radiation oncology, and most importantly, assigning a patient navigator to coordinate care and serve as the liaison between patients, physicians, and schedulers.^18^ Another project reduced preventable delays from 24% to 9% and improved overall timeliness of care from 62% to 73% by initiating dental evaluation at the time of the new patient visit with scheduled extraction during surgery, placing radiation oncology referrals at the time of identified indication for PORT, setting automated reminders to review surgical pathology if there was a clinical concern for PORT, and creating a standardized head and neck oncology clinic visit checklist to recap key aspects of the care timeline.^19^ The most successful intervention to date, Navigation for Disparities and Untimely Radiation thErapy (NDURE), involves a series of three appointments with a patient navigator throughout the treatment course to coordinate care, provide patient education, and identify and resolve potential modifiable barriers to PORT. This resulted in 86% of patients receiving PORT within 6 weeks; however, it should be noted that there were only 15 patients in total in the study.^20^ Additionally, nomograms and machine learning algorithms are available to identify patients at highest risk for PORT delay.^21,22^

Prolonged length of stay and unplanned hospital readmissions are often due to surgical complications. Therefore, efforts to minimize complications via emphasis on measures such as adequate preoperative nutrition, medical optimization, and thromboprophylaxis may be useful. Importantly, multidisciplinary communication is critical in advance of surgery. Patients should either see their radiation oncologist preoperatively or have a postoperative appointment scheduled in advance. This may help avoid last minute care coordination and delays if a patient desires surgical and radiation care at different facilities for example, a variable that was significantly associated with delay in the current study. It is essential that institutions managing HNSCC patients evaluate and implement similar interventions to improve adherence to national guidelines.

A distinct advantage of TriNetX is analysis of additional factors that were not studied previously. Specifically, patients who underwent a neck dissection, certain free flaps, or laryngectomy were more likely to experience PORT delay. This also applied to those with a tracheostomy or gastrostomy tube. This suggests patients with more extensive surgeries or in need of additional ancillary services are at increased risk for PORT delay. Additionally, patients in TriNetX who were never married, divorced, or widowed were significantly more likely to receive PORT beyond 6 weeks. A possible explanation for this could be that partners were likely to provide tangible services and informal caregiving, such as transportation and care coordination that resulted in increased adherence to guidelines. The improved timeliness of treatment in the married cohort may also be extrapolated to any unmarried patients with caring partners who will provide various levels of support after surgery. Additionally, other family members, friends, neighbors, or patient navigators may provide a similar positive influence. Initiatives aimed at providing these conveniences may be worth pursuing in patients lacking support.

Now that time to PORT is officially a CoC national quality metric,^7^ head and neck oncologists and other members of the multidisciplinary care team will prioritize similar efforts to improve adherence. Our results provide a performance baseline, with future research necessary to continue trending outcomes moving forward. While our study provides a recent update regarding adherence to treatment guidelines and is the first to identify certain factors as predictive of delay, it is not without limitations. First, it is limited by the quality of data entry in the NCDB and TriNetX, which is a recognized weakness in performing a retrospective review and using a large de-identified database. However, the similar rate of treatment delay in two databases increased our confidence in our findings, and the large sample size of patients strengthened the generalizability. Furthermore, TriNetX was only able to capture those patients who received both surgery and radiation within the same HCO, which likely excluded a significant number of patients, but these patients with fragmented care were able to be included in the analysis of the NCDB. It is possible that there could be some patient overlap between databases, however the second database was useful in that it allowed for the presentation of more granular data. A limitation affecting our analysis of both databases is that we were unable to determine whether patients were subject to circumstances that justifiably delayed adjuvant radiation, which may have inflated our reported rate of delay. Components that are recognized as leading to prolonged time to PORT, such as delays in dental evaluation, necessary treatment preradiation, and transportation issues, were also not able to be captured through these databases.

In conclusion, timely initiation of PORT continues to be a challenge for nearly two-thirds of patients with HNSCC. With the introduction of a head and neck specific CoC quality measure evaluating this benchmark, we anticipate renewed institutional focus on vulnerable patients and care processes associated with delay. A lack of family support, more complex surgical care, and demographic factors associated with poorer access to care continue to be risk factors for delayed adjuvant radiation.

## Supplementary Material

supplemental file

## Figures and Tables

**TABLE 1:** NCDB analysis of demographic characteristics

Patient variable	Total patients (*n* = 40 164)	Initiation of PORT ≤6 weeks (*n* = 15 263)	Initiation of PORT >6 weeks (*n* = 24 901)	OR [95% CI]
Age				
<50	5258	2122 (40.4%)	3136 (59.6%)	1.00 [Ref]
50–59	11 424	4296 (37.6%)	7128 (62.4%)	1.12 [1.05–1.20]
60–69	13 397	5063 (37.8%)	8334 (62.2%)	1.11 [1.04–1.18]
>70	10 085	3782 (37.5%)	6303 (62.5%)	1.28 [1.05–1.20]
Sex				
Male	29 739	11 858 (39.9%)	17 881 (60.1%)	1.00 [Ref]
Female	10 425	3405 (32.7%)	7020 (67.3%)	1.36 [1.30–1.43]
Race				
White	35 080	13 722 (39.1%)	21 358 (60.9%)	1.00 [Ref]
Black	3130	929 (29.7%)	2201 (70.3%)	1.52 [1.40–1.64]
Asian/Pacific Islander	1193	361 (30.3%)	832 (69.7%)	1.48 [1.30–1.67]
Other/unknown	761	251 (33.0%)	510 (67.0%)	1.30 [1.12–1.52]
Ethnicity				
Non-Hispanic	37 629	14 408 (38.3%)	23 221 (61.7%)	1.00 [Ref]
Hispanic	1862	600 (32.2%)	1262 (67.8%)	1.30 [1.18–1.44]
Unknown	673	255 (37.9%)	418 (62.1%)	1.01 [0.86–1.19]
Insurance status				
Private insurance	1140	344 (30.2%)	796 (69.8%)	1.00 [Ref]
Not insured	17 418	7529 (43.2%)	9889 (56.8%)	1.76 [1.54–2.00]
Medicaid	4591	1216 (26.5%)	3375 (73.5%)	2.11 [1.96–2.27]
Medicare	15 551	5644 (36.3%)	9907 (63.7%)	1.33 [1.27–1.39]
Other government	983	316 (32.1%)	667 (67.9%)	1.60 [1.40–1.84]
Unknown	481	214 (44.5%)	267 (55.5%)	0.95 [0.79–1.14]
Area of residence^a^				
Metropolitan	32 187	12 150 (37.7%)	20 037 (62.3%)	1.00 [Ref]
Urban	5899	2317 (39.3%)	3582 (60.7%)	0.93 [0.87–0.99]
Rural	686	264 (38.5%)	422 (61.5%)	0.93 [0.78–1.10]
Educational attainment^a^				
Highest quartile	8386	3571 (42.6%)	4815 (57.4%)	1.00 [Ref]
Second highest quartile	9818	3782 (38.5%)	6036 (61.5%)	1.11 [1.03–1.20]
Second lowest quartile	9057	3252 (35.9%)	5805 (64.1%)	1.27 [1.18–1.37]
Lowest quartile	6711	2221 (33.1%)	4490 (66.9%)	1.54 [1.43–1.67]
Median household income^a^				
Less than $40227	6225	2088 (33.5%)	4137 (66.5%)	1.00 [Ref]
$40227–$50353	7660	2809 (36.7%)	4851 (63.3%)	0.87 [0.81–0.93]
$50354–$63332	7750	2963 (38.2%)	4787 (61.8%)	0.81 [0.76–0.87]
$63333+	12 260	4941 (40.3%)	7319 (59.7%)	0.74 [0.70–0.79]
Distance to hospital (miles)				
≤10	19 724	7686 (39.0%)	12 038 (61.0%)	1.00 [Ref]
11–20	6669	2593 (38.9%)	4076 (61.1%)	1.00 [0.94–1.06]
21–50	7640	2810 (36.8%)	4830 (63.2%)	1.09 [1.03–1.15]
51–10	3683	1232 (33.5%)	2451 (66.5%)	1.27 [1.17–1.36]
>100	2448	942 (38.5%)	1506 (61.5%)	1.02 [0.93–1.11]

Abbreviations: CI, confidence interval; OR, odds ratio; PORT, postoperative radiation therapy; Ref, reference category.

a Certain columns may not sum to the total due to missing observations.

**TABLE 2:** NCDB and TriNetX time to PORT breakdown

Commencement of PORT	NCDB (*n* = 40 164)	NCDB cumulative frequency	TriNetX (*n* = 3165)	TriNetX cumulative frequency
≤6 weeks	15 263 (38.0%)	15 263 (38.0%)	1142 (36.1%)	1142 (36.1%)
6–8 weeks	12 169 (30.3%)	27 432 (68.3%)	931 (29.4%)	2073 (65.5%)
8–10 weeks	6327 (15.8%)	33 759 (84.1%)	491 (15.5%)	2564 (81.0%)
10–12 weeks	2993 (7.5%)	36 752 (91.5%)	244 (7.7%)	2808 (88.7%)
>12 weeks	3412 (8.5%)	40 164 (100%)	357 (11.3%)	3165 (100%)

Abbreviations: NCDB, National Cancer Database; PORT, postoperative radiation therapy.

**TABLE 3:** NCDB analysis of clinical and surgical characteristics

Patient variable	Total patients (*n* = 40 164)	Initiation of PORT ≤6 weeks (*n* = 15 263)	Initiation of PORT >6 weeks (*n* = 24 901)	OR [95% CI]
Cancer primary site				
Oropharynx	15 099	7198 (47.7%)	7901 (52.3%)	1.00 [Ref]
Oral cavity	16 415	4297 (26.2%)	12 118 (73.8%)	2.56 [2.45–2.69]
Hypopharynx	896	304 (33.9%)	592 (66.1%)	1.77 [1.53–2.04]
Larynx	7754	3464 (44.7%)	4290 (55.3%)	1.12 [1.06–1.19]
AJCC clinical stage group^a^				
I	2719	1354 (49.8%)	1365 (50.2%)	1.00 [Ref]
II	2766	985 (35.6%)	1781 (64.4%)	1.79 [1.61–1.99]
III	3677	1457 (39.6%)	2220 (60.4%)	1.51 [1.36–1.67]
IV	10 468	3886 (37.1%)	6582 (62.9%)	1.68 [1.54–1.82]
Unknown	126	48 (38.1%)	78 (61.9%)	1.61 [1.11–2.32]
AJCC pathological stage group^a^				
I	1126	485 (43.1%)	641 (56.9%)	1.00 [Ref]
II	1406	468 (33.3%)	938 (66.7%)	1.51 [1.29–1.78]
III	3044	1083 (35.6%)	1961 (64.4%)	1.37 [1.19–1.57]
IV	29 015	10 084 (34.8%)	18 931 (65.2%)	1.42 [1.25–1.60]
Surgical margin status^a^				
Negative	26 698	8873 (33.2%)	17 825 (66.8%)	1.00 [Ref]
Positive	10 596	4811 (45.4%)	5785 (54.6%)	0.59 [0.57–0.62]
Unknown	2870	1579 (55.0%)	1291 (45.0%)	0.40 [0.37–0.44]
Postoperative length of stay (days)				
0–3	18 596	9205 (49.5%)	9391 (50.5%)	1.00 [Ref]
4–7	7012	2273 (32.4%)	4739 (67.6%)	1.78 [1.67–1.90]
8–14	7664	1928 (25.2%)	5736 (74.8%)	2.04 [1.92–2.16]
15–21	1186	155 (13.1%)	1031 (86.9%)	6.51 [5.49–7.73]
>21	1042	132 (12.7%)	910 (87.3%)	6.75 [5.61–8.12]
Unknown	4664	1570 (33.7%)	3094 (66.3%)	1.93 [1.80–2.06]
Readmission within 30 days of discharge				
None	37 474	14 428 (38.5%)	23 046 (61.5%)	1.00 [Ref]
Unplanned	1078	278 (25.8%)	800 (74.2%)	1.80 [1.56–2.06]
Planned	726	269 (37.1%)	457 (62.9%)	1.06 [0.91–1.23]
Unknown	886	288 (32.5%)	598 (67.5%)	1.30 [1.12–1.49]
Radiation modality^a^				
External beam	2005	1007 (50.2%)	998 (49.8%)	1.00 [Ref]
Conformal or 3D therapy	11 774	4767 (40.5%)	7007 (59.5%)	0.67 [0.61–0.74]
IMRT	25 843	9296 (36.0%)	16 547 (64.0%)	1.21 [1.15–1.26]
Concurrent chemoradiation				
No	30 062	10 946 (36.4%)	19 116 (63.6%)	1.00 [Ref]
Yes	10 102	4317 (42.7%)	5785 (57.3%)	0.76 [0.73–0.80]
Charlson-Deyo comorbidity score				
0	29 888	11 641 (38.9%)	18 247 (61.1%)	1.00 [Ref]
1	6765	2390 (35.3%)	4375 (64.7%)	1.16 [1.10–1.23]
≥2	3511	1232 (35.1%)	2279 (64.9%)	1.18 [1.09–1.27]

Abbreviations: AJCC, American Joint Committee on Cancer; CI, confidence interval; IMRT, intensity-modulated radiation therapy; OR, odds ratio; PORT, postoperative radiation therapy; Ref, reference category; 3D, 3-dimensional.

a Certain columns may not sum to the total due to missing observations.

**TABLE 4:** NCDB analysis of hospital characteristics

Patient variable	Total patients (*n* = 40 164)	Initiation of PORT ≤6 weeks (*n* = 15 263)	Initiation of PORT >6 weeks (*n* = 24 901)	OR [95% CI]
Treatment facility type^a^				
Community Cancer Program	1909	797 (41.7%)	1112 (58.3%)	1.00 [Ref]
Comprehensive Community Cancer Program	10 436	4524 (43.3%)	5912 (56.7%)	0.93 [0.84–1.03]
Academic/Research Program	20 557	6905 (33.6%)	13 652 (66.4%)	1.41 [1.28–1.55]
Integrated Network Cancer Program	6165	2606 (42.3%)	3559 (57.7%)	0.97 [0.88–1.08]
Number of facilities involved in treatment				
All treatment at 1 CoC Facility	30 255	11 823 (39.1%)	18 432 (60.9%)	1.00 [Ref]
Treatment at >1 CoC Facility	9909	3440 (34.7%)	6469 (65.3%)	1.20 [1.15–1.26]
Surgery and radiation at same facility				
Yes	20 312	7024 (34.6%)	13 288 (65.4%)	1.00 [Ref]
No	19 852	8239 (41.5%)	11 613 (58.5%)	1.34 [1.28–1.39]
Region of care^a^				
Northeast	8070	2702 (33.5%)	5368 (66.5%)	1.00 [Ref]
Midwest	11 014	4604 (41.8%)	6410 (48.2%)	0.70 [0.66–0.74]
South	14 025	5296 (37.8%)	8729 (62.2%)	0.83 [0.78–0.87]
West	5958	2230 (37.4%)	3728 (62.6%)	0.84 [0.78–0.90]

Abbreviations: CI, confidence interval; CoC, commission on cancer; OR, odds ratio; PORT, postoperative radiation therapy; Ref, reference category.

a Certain columns may not sum to the total due to missing observations.

**TABLE 5:** Risk factors for prolonged length of stay

	Postoperative length of stay (days)					
Patient variable	0–3 (*n* = 18 596)	4–7 (*n* = 7012)	8–14 (*n* = 7664)	15–21 (*n* = 1186)	>21 (*n* = 1042)	*p*-value
Age						
<50	2392 (51.4%)	1006 (21.6%)	1016 (21.8%)	137 (2.9%)	106 (2.3%)	<0.001^a^
50–59	5381 (53.0%)	2018 (19.9%)	2091 (20.6%)	336 (3.3%)	321 (3.2%)	
60–69	6062 (51.6%)	2391 (20.3%)	2567 (21.8%)	396 (3.4%)	340 (2.9%)	
>70	4761 (52.3%)	1597 (17.9%)	1990 (22.3%)	317 (3.5%)	275 (3.1%)	
Sex						
Male	14 291 (54.5%)	5069 (19.3%)	5295 (20.2%)	817 (3.1%)	739 (2.8%)	<0.001^a^
Female	4305 (46.3%)	1943 (20.9%)	2369 (25.5%)	369 (4.0%)	303 (3.3%)	
Race						
White	16 856 (54.4%)	6117 (19.7%)	6243 (20.2%)	931 (3.0%)	826 (2.7%)	<0.001^a^
Black	1072 (38.4%)	523 (18.7%)	878 (31.5%)	176 (6.3%)	142 (5.1%)	
Asian/Pacific Islander	372 (34.7%)	228 (21.3%)	367 (34.2%)	62 (5.8%)	43 (4.0%)	
Other/unknown	296 (44.6%)	144 (21.7%)	176 (26.5%)	17 (2.6%)	31 (4.7%)	
Ethnicity						
Non-Hispanic	17 538 (52.7%)	6566 (19.7%)	7095 (21.3%)	1106 (3.3%)	957 (2.9%)	<0.001^a^
Hispanic	737 (43.8%)	348 (20.7%)	464 (27.6%)	63 (3.7%)	71 (4.2%)	
Unknown	321 (57.8%)	98 (17.7%)	105 (18.9%)	17 (3.1%)	14 (2.5%)	
Insurance status						
Not insured	383 (37.4%)	227 (22.1%)	323 (31.5%)	46 (4.5%)	46 (4.5%)	<0.001^a^
Private insurance	9049 (58.5%)	3123 (20.2%)	2700 (17.5%)	334 (2.2%)	259 (1.7%)	
Medicaid	1424 (34.7%)	875 (21.4%)	1305 (31.8%)	239 (5.8%)	255 (6.2%)	
Medicare	7126 (51.8%)	2545 (18.5%)	3118 (22.7%)	518 (3.8%)	450 (3.3%)	
Other government	398 (53.1%)	152 (20.3%)	146 (19.5%)	33 (4.4%)	21 (2.8%)	
Insurance status unknown	216 (53.3%)	90 (22.2%)	72 (1.8%)	16 (4.0%)	11 (2.7%)	
Urban/rural						
Metropolitan	15 006 (52.7%)	5525 (19.4%)	6115 (21.5%)	974 (3.4%)	853 (3.0%)	0.01^a^
Urban	2679 (52.1%)	1063 (20.7%)	1101 (21.4%)	149 (2.9%)	151 (2.9%)	
Rural	289 (48.3%)	137 (22.9%)	139 (23.2%)	16 (2.7%)	17 (2.8%)	
Education						
Lowest quartile	2707 (45.9%)	1131 (19.2%)	1530 (25.9%)	279 (4.7%)	250 (4.2%)	<0.001^a^
Second lowest quartile	4010 (50.6%)	1595 (20.1%)	1807 (22.8%)	295 (3.7%)	216 (2.7%)	
Second highest quartile	4661 (53.9%)	1671 (19.3%)	1795 (20.8%)	267 (3.1%)	250 (2.9%)	
Highest quartile	4260 (56.9%)	1499 (20.0%)	1391 (18.6%)	182 (2.4%)	149 (2.0%)	
Median household income						
Less than $40227	2483 (45.5%)	1067 (19.6%)	1432 (26.3%)	252 (4.6%)	221 (4.1%)	<0.001^a^
$40227–$50353	3380 (50.9%)	1358 (20.5%)	1500 (22.6%)	220 (3.3%)	181 (2.7%)	
$50354–$63332	3564 (52.2%)	1336 (19.6%)	1479 (21.7%)	241 (3.5%)	203 (3.0%)	
$63333+	6176 (56.4%)	2126 (19.4%)	2096 (19.1%)	307 (2.8%)	255 (2.3%)	
Great circle distance						
≤10	9843 (57.2%)	29,12 (16.9%)	3367 (19.6%)	569 (3.3%)	511 (3.0%)	<0.001^a^
11–20	3251 (56.0%)	1049 (18.1%)	1158 (20.0%)	190 (3.3%)	153 (2.6%)	
21–50	3327 (48.9%)	1472 (21.6%)	1585 (23.3%)	221 (3.2%)	198 (2.9%)	
51–100	1386 (40.1%)	896 (25.9%)	928 (26.9%)	124 (3.6%)	120 (3.5%)	
>100	789 (35.2%)	683 (30.5%)	626 (27.9%)	82 (3.7%)	60 (2.7%)	
Charlson–Deyo score						
0	14 406 (55.1%)	5108 (19.5%)	5207 (19.9%)	767 (2.9%)	666 (2.5%)	<0.001^a^
1	2816 (45.8%)	1260 (20.5%)	1589 (25.9%)	263 (4.3%)	217 (3.5%)	
≥2	1374 (42.9%)	644 (20.1%)	868 (27.1%)	156 (4.9%)	159 (5.0%)	
Primary site						
Oral cavity	4653 (31.8%)	3611 (24.7%)	4933 (33.7%)	816 (5.6%)	630 (4.3%)	<0.001^a^
Oropharynx	9981 (75.2%)	2099 (15.8%)	908 (6.8%)	133 (1.0%)	148 (1.1%)	
Hypopharynx	253 (32.3%)	168 (21.4%)	270 (34.4%)	41 (5.2%)	52 (6.6%)	
Larynx	3709 (54.5%)	1134 (16.7%)	1553 (22.8%)	196 (2.9%)	212 (3.1%)	
AJCC clinical stage group						
1	1963 (81.6%)	237 (9.9%)	168 (7.0%)	18 (0.7%)	20 (0.8%)	<0.001^a^
2	1307 (52.7%)	553 (22.3%)	500 (20.2%)	64 (2.6%)	57 (2.3%)	
3	1692 (52.0%)	752 (23.1%)	651 (20.0%)	88 (2.7%)	72 (2.2%)	
4	4041 (43.6%)	2071 (22.4%)	2405 (26.0%)	387 (4.2%)	358 (3.9%)	
Unknown	55 (52.9%)	20 (19.2%)	20 (19.2%)	6 (5.8%)	3 (2.9%)	
AJCC pathological stage group						
1	753 (75.1%)	139 (13.9%)	89 (8.9%)	7 (0.7%)	15 (1.5%)	<0.001^a^
2	582 (47.0%)	315 (25.4%)	273 (22.0%)	39 (3.1%)	30 (2.4%)	
3	1291 (47.4%)	649 (23.8%)	639 (23.5%)	83 (3.0%)	60 (2.2%)	
4	11 614 (45.1%)	5697 (22.1%)	6527 (25.3%)	1032 (4.0%)	907 (3.5%)	
Surgical margin status						
Negative	10 154 (42.5%)	5761 (24.1%)	6279 (26.3%)	927 (3.9%)	771 (3.2%)	<0.001^a^
Positive	6251 (68.0%)	1145 (12.5%)	1300 (14.1%)	245 (2.7%)	253 (2.8%)	
Unknown	2191 (90.8%)	106 (4.4%)	85 (3.5%)	14 (0.6%)	18 (0.7%)	
Readmission within 30 days of discharge						
None	17 683 (53.0%)	6514 (19.5%)	7137 (21.4%)	1080 (3.2%)	950 (2.8%)	<0.001^a^
Unplanned	306 (28.8%)	291 (27.4%)	326 (30.7%)	77 (7.3%)	61 (5.7%)	
Planned	362 (51.6%)	146 (20.8%)	149 (21.2%)	24 (3.4%)	21 (3.0%)	
Unknown	245 (65.7%)	61 (16.4%)	52 (13.9%)	5 (1.3%)	10 (2.7%)	
Radiation modality						
Conformal or 3D therapy	1302 (74.7%)	193 (11.1%)	185 (10.6%)	35 (2.0%)	29 (1.7%)	<0.001^a^
External beam	5679 (54.4%)	2032 (19.5%)	2133 (20.4%)	333 (3.2%)	265 (2.5%)	
IMRT	11 314 (49.5%)	4715 (20.6%)	5269 (23.1%)	805 (3.5%)	734 (3.2%)	
Concurrent chemoradiation						
No	13 834 (52.0%)	5320 (20.0%)	5760 (21.7%)	899 (3.4%)	788 (3.0%)	0.14^a^
Yes	4762 (53.5%)	1692 (19.0%)	1904 (21.4%)	287 (3.2%)	254 (2.9%)	

Abbreviations: AJCC, American Joint Committee on Cancer; IMRT, intensity-modulated radiation therapy; 3D, 3-dimensional.

a Chi-square *p*-value.

**TABLE 6:** Risk factors for hospital readmission

	Readmission within 30 days of discharge				
Patient variable	None (*n* = 37 474)	Unplanned (*n* = 1078)	Planned (*n* = 726)	Unknown (*n* = 886)	*p*-value
Age					
<50	4870 (92.6%)	164 (3.1%)	106 (2.0%)	118 (2.2%)	0.14^a^
50–59	10 656 (93.3%)	320 (2.8%)	210 (1.8%)	238 (2.1%)	
60–69	12 494 (93.3%)	343 (2.6%)	253 (1.9%)	307 (2.3%)	
>70	9454 (93.7%)	251 (2.5%)	157 (1.6%)	223 (2.2%)	
Sex					
Male	27 766 (93.4%)	789 (2.7%)	533 (1.8%)	651 (2.2%)	0.86^a^
Female	9708 (93.1%)	289 (2.8%)	193 (1.9%)	235 (2.3%)	
Race					
White	32 794 (93.5%)	911 (2.6%)	612 (1.7%)	763 (2.2%)	<0.001^a^
Black	2878 (91.9%)	118 (3.8%)	65 (2.1%)	69 (2.2%)	
Asian/Pacific Islander	1105 (92.6%)	30 (2.5%)	31 (2.6%)	27 (2.3%)	
Other/unknown	697 (91.6%)	19 (2.5%)	18 (2.4%)	27 (3.5%)	
Ethnicity					
Non-Hispanic	35 092 (93.3%)	1028 (2.7%)	690 (1.8%)	819 (2.2%)	0.10^a^
Hispanic	1751 (94.0%)	36 (1.9%)	28 (1.5%)	47 (2.5%)	
Unknown	631 (93.8%)	14 (2.1%)	8 (1.2%)	20 (3.0%)	
Insurance status					
Not insured	1067 (93.6%)	27 (2.4%)	29 (2.5%)	17 (1.5%)	<0.001^a^
Private insurance	16 332 (93.8%)	412 (2.4%)	323 (1.9%)	351 (2.0%)	
Medicaid	4257 (92.7%)	152 (3.3%)	77 (1.7%)	105 (2.3%)	
Medicare	14 499 (93.2%)	453 (2.9%)	259 (1.7%)	340 (2.2%)	
Other government	895 (91.0%)	23 (2.3%)	17 (1.7%)	48 (4.9%)	
Insurance status unknown	424 (88.1%)	11 (2.3%)	21 (4.4%)	25 (5.2%)	
Urban/rural					
Metropolitan	30 038 (93.3%)	880 (2.7%)	607 (1.9%)	662 (2.1%)	<0.001^a^
Urban	5475 (92.8%)	148 (2.5%)	100 (1.7%)	176 (3.0%)	
Rural	633 (92.3%)	23 (3.4%)	8 (1.2%)	22 (3.2%)	
Education					
Lowest quartile	6213 (92.6%)	209 (3.1%)	125 (1.9%)	164 (2.4%)	0.01^a^
Second lowest quartile	8451 (93.3%)	238 (2.6%)	148 (1.6%)	220 (2.4%)	
Second highest quartile	9183 (93.5%)	239 (2.4%)	181 (1.8%)	215 (2.2%)	
Highest quartile	7855 (93.7%)	222 (2.6%)	161 (1.9%)	148 (1.8%)	
Median household income					
Less than $40227	5734 (92.1%)	206 (3.3%)	113 (1.8%)	172 (2.8%)	0.001^a^
$40227–$50353	7181 (93.7%)	183 (2.4%)	130 (1.7%)	166 (2.2%)	
$50354–$63332	7264 (93.7%)	195 (2.5%)	134 (1.7%)	157 (2.0%)	
$63333+	11 450 (93.4%)	322 (2.6%)	238 (1.9%)	250 (2.0%)	
Great circle distance					
≤10	18 298 (92.8%)	519 (2.6%)	362 (1.8%)	545 (2.8%)	<0.001^a^
11–20	6237 (93.5%)	161 (2.4%)	120 (1.8%)	151 (2.3%)	
21–50	7191 (94.1%)	214 (2.8%)	118 (1.5%)	117 (1.5%)	
51–100	3458 (93.9%)	106 (2.9%)	80 (2.2%)	39 (1.1%)	
>100	2290 (93.5%)	78 (3.2%)	46 (1.9%)	34 (1.4%)	
Charlson–Deyo score					
0	27 911 (93.4%)	719 (2.4%)	529 (1.8%)	729 (2.4%)	<0.001^a^
1	6302 (93.2%)	220 (3.3%)	129 (1.9%)	114 (1.7%)	
≥2	3261 (92.9%)	139 (4.0%)	68 (1.9%)	43 (1.2%)	
Primary site					
Oral cavity	15 227 (92.8%)	533 (3.2%)	294 (1.8%)	361 (2.2%)	<0.001^a^
Oropharynx	14 154 (93.7%)	323 (2.1%)	279 (1.8%)	343 (2.3%)	
Hypopharynx	812 (90.6%)	38 (4.2%)	17 (1.9%)	29 (3.2%)	
Larynx	7281 (93.9%)	184 (2.3%)	136 (1.7%)	153 (2.0%)	
AJCC clinical stage group					
1	2594 (95.4%)	32 (1.2%)	48 (1.8%)	45 (1.7%)	<0.001^a^
2	2610 (94.4%)	63 (2.3%)	51 (1.8%)	42 (1.5%)	
3	3444 (93.7%)	99 (2.7%)	62 (1.7%)	72 (2.0%)	
4	9733 (93.0%)	308 (2.9%)	216 (2.1%)	211 (2.0%)	
Unknown	117 (92.9%)	3 (2.4%)	4 (3.2%)	2 (1.6%)	
AJCC pathological stage group					
0	110 (96.5%)	1 (0.9%)	2 (1.8%)	1 (0.9%)	0.02^a^
1	1072 (95.2%)	17 (1.5%)	18 (1.6%)	19 (1.7%)	
2	1335 (95.0%)	30 (2.1%)	20 (1.4%)	21 (1.5%)	
3	2819 (92.6%)	84 (2.8%)	62 (2.0%)	79 (2.6%)	
4	26 946 (92.9%)	866 (3.0%)	547 (1.9%)	656 (2.3%)	
Surgical margin status					
Negative	24 882 (93.2%)	790 (3.0%)	506 (1.9%)	520 (1.9%)	<0.001^a^
Positive	9904 (93.5%)	261 (2.5%)	184 (1.7%)	247 (2.3%)	
Unknown	2688 (93.7%)	27 (0.9%)	36 (1.3%)	119 (4.1%)	
Postoperative length of stay (days)					
0–3	17 683 (95.1%)	306 (1.6%)	362 (1.9%)	245 (1.3%)	<0.001^a^
4–7	6514 (92.9%)	291 (4.2%)	146 (2.1%)	61 (0.9%)	
8–14	7137 (93.1%)	326 (4.3%)	149 (1.9%)	52 (0.7%)	
15–21	1080 (91.1%)	77 (6.5%)	24 (2.0%)	5 (0.4%)	
>21	950 (91.2%)	61 (5.9%)	21 (2.0%)	10 (1.0%)	
Radiation modality					
Conformal or 3D therapy	1902 (94.9%)	40 (2.0%)	24 (1.2%)	39 (1.9%)	0.003^a^
External beam	11 044 (93.8%)	291 (2.5%)	200 (1.7%)	239 (2.0%)	
IMRT	24 007 (92.9%)	743 (2.9%)	494 (1.9%)	599 (2.3%)	
Concurrent chemoradiation					
No	28 048 (93.3%)	825 (2.7%)	534 (1.8%)	655 (2.2%)	0.45^a^
Yes	9426 (93.3%)	253 (2.5%)	192 (1.9%)	231 (2.3%)	

Abbreviations: AJCC, American Joint Committee on Cancer; IMRT, intensity-modulated radiation therapy; 3D, 3-dimensional.

a Chi-square *p*-value.

**TABLE 7:** TriNetX analysis of demographic characteristics

Patient variable	Total patients (*n* = 3165)	Initiation of PORT ≤6 weeks (*n* = 1142)	Initiation of PORT >6 weeks (*n* = 2023)	OR [95% CI]
Age				
<65 years	1570	564 (35.9%)	1006 (64.1%)	1 [Ref]
≥65 years	1595	578 (36.2%)	1017 (63.8%)	0.99 [0.85–1.01]
Sex				
Male	2331	874 (37.5%)	1457 (62.5%)	1 [Ref]
Female	834	268 (32.1%)	566 (67.9%)	1.27 [1.08–1.49]
Race				
White	2554	937 (36.7%)	1617 (63.3%)	1 [Ref]
Black	387	131 (33.9%)	256 (66.1%)	1.14 [0.91–1.43]
Asian	57	21 (36.8%)	36 (63.2%)	0.99 [0.58–1.72]
Other/unknown	167	53 (31.7%)	114 (68.3%)	1.25 [0.89–1.75]
Ethnicity				
Non-Hispanic or Latino	2622	969 (37.0%)	1653 (63.0%)	1 [Ref]
Hispanic or Latino	179	62 (34.6%)	117 (65.4%)	1.11 [0.81–1.52]
Other/unknown	364	111 (30.5%)	253 (69.5%)	1.33 [1.05–1.69]
Marital status				
Married	966	422 (43.7%)	544 (56.3%)	1 [Ref]
Never married	418	142 (34.0%)	276 (66.0%)	1.51 [1.19–1.92]
Divorced	194	69 (35.6%)	125 (64.4%)	1.41 [1.02–1.92]
Widowed	133	46 (34.6%)	87 (65.4%)	1.47 [1.01–2.13]
Other/unknown	1454	463 (31.8%)	991 (68.2%)	1.67 [1.41–1.96]
Region of care				
East	2041	731 (35.8%)	1310 (64.2%)	1 [Ref]
West	1112	408 (36.7%)	704 (63.3%)	0.96 [0.83–1.12]
Unknown	12	3 (25.0%)	9 (75.0%)	1.67 [0.45–6.25]

Abbreviations: CI, confidence interval; OR, odds ratio; PORT, postoperative radiation therapy; Ref, reference category.

**TABLE 8:** TriNetX analysis of clinical and surgical characteristics

Patient variable	Total patients (*n* = 3165)	Initiation of PORT ≤6 weeks (*n* = 1142)	Initiation of PORT >6 weeks (*n* = 2023)	OR [95% CI]
Cancer site				
Oropharynx	1116	485 (43.5%)	631 (56.5%)	1 [Ref]
Oral cavity	1376	412 (29.9%)	964 (70.1%)	1.79 [1.52–2.12]
Hypopharynx	98	46 (46.9%)	52 (53.1%)	0.87 [0.57–1.31]
Larynx	575	199 (34.6%)	376 (65.4%)	1.45 [1.18–1.79]
Concurrent chemoradiation				
No	1947	660 (33.9%)	1287 (66.1%)	1 [Ref]
Yes	1218	482 (39.6%)	736 (60.4%)	0.78 [0.68–0.91]
Neck dissection				
No	1387	579 (41.7%)	808 (58.3%)	1 [Ref]
Yes	1778	563 (31.7%)	1215 (68.3%)	1.55 [1.34–1.79]
Free skin flap				
No	2755	997 (36.2%)	1758 (63.8%)	1 [Ref]
Yes	410	145 (35.4%)	265 (64.6%)	1.04 [0.83–1.29]
Free osteocutaneous flap				
No	2839	1057 (37.2%)	1782 (62.8%)	1 [Ref]
Yes	326	85 (26.1%)	241 (73.9%)	1.68 [1.30–2.18]
Free muscle or myocutaneous flap				
No	3008	1113 (37.0%)	1895 (63.0%)	1 [Ref]
Yes	157	29 (18.5%)	128 (81.5%)	2.59 [1.72–3.91]
Free fascial flap				
No	3081	1125 (37.0%)	1956 (63.0%)	1 [Ref]
Yes	84	17 (20.2%)	67 (79.8%)	2.27 [1.32–3.88]
Laryngectomy				
No	2839	1067 (37.6%)	1772 (62.4%)	1 [Ref]
Yes	326	75 (23.0%)	251 (77.0%)	2.01 [1.54–2.64]
Gastrostomy status				
No	2030	776 (38.2%)	1254 (61.8%)	1 [Ref]
Yes	1135	366 (32.2%)	769 (67.8%)	1.30 [1.11–1.51]
Tracheostomy status				
No	2234	880 (39.4%)	1354 (60.6%)	1 [Ref]
Yes	931	262 (28.1%)	669 (71.9%)	1.67 [1.41–1.96]

Abbreviations: CI, confidence interval; OR, odds ratio; PORT, postoperative radiation therapy; Ref, reference category.

## Data Availability

The study data is available from F. Jeffrey Lorenz on reasonable request.
