# Supplementary material for: Update on adherence to guidelines for time to initiation of postoperative radiation for head and neck squamous cell carcinoma
Source: Head Neck. Author manuscript; Available in PMC 2024 Jan 19. (PMC10797635; doi:10.1002/hed.27380)
Supplement: supplemental file [file NIHMS1953199-supplement-supplemental_file.docx]

**Supplement:**

Supplement 1: Diagnosis and Procedure Codes

**Head and neck cancer subsites:**

Oropharynx: C01, C02.4, C05.1, C05.2, C05.8, C05.9, C09.0, C09.1, C09.8, C09.9, C10.0, C10.1, C10.2, C10.3, C10.4, C10.8, C10.9, C14.0, C14.2, C14.8

Oral Cavity: C00.0, C00.1, C00.2, C00.3, C00.4, C00.5, C00.6, C00.8, C00.9, C02.0, C02.1, C02.2, C02.3, C02.8, C02.9, C03.0, C03.1, C03.9, C04.0, C04.1, C04.8, C04.9, C05.0, C06.0, C06.1, C06.2, C06.8, C06.9

Larynx: C32.1, C32.2, C32.3, C32.0, C32.8, C32.9

Hypopharynx: C12, C13.0, C13.1, C13.2, C13.8, C13.9

Surgical resection: 1005785, 1005815, 1006969, 1006999, 1007040, 1007046, 1007076, 1007093, 1007190, 1031037, 15756, 15757, 15758, 20955, 38700, 38720, 38724, 40530, 41110, 41116, 41820, 41830, 42120, 42140, 42808, 42826, 42870, 42890, 42892, 42894, 07B1, 07B2, 09BN, 09TN, 0CB0, 0CB1, 0CB2, 0CB3, 0CB4, 0CB5, 0CB6, 0CB7, 0CBM, 0CBP, 0CBR, 0CBS, 0CBT, 0CBV, 0CT0, 0CT1, 0CT2, 0CT3, 0CT7, 0CTM, 0CTP, 0CTR, 0CTS, 0CTT, 0CTV, 0NBR, 0NBT, 0NBV, 0NTR, 0NTT, 0NTV

Radiation: 77385, 77386, 77401, 77402, 77407, 77412, 77520, 77522, 77523, 77525, G6004, G6005, G6006, G6007, G6008, G6009, G6010, G6011, G6012, G6013, G6014, G6015, G6016,

Stereotactic radiosurgery: 77371, 77372, 77373

Brachytherapy: 1010919

**Clinical and surgical variables:**

Chemotherapy: 1013438

Neck dissection: 1006913

Free skin flap: 15757

Free osteocutaneous flap: 1003777

Free muscle or myocutaneous flap: 15756

Free fascial flap: 15758

Laryngectomy: 1005815

Gastrostomy status: Z93.1

Tracheostomy status: Z93.0

Supplement 2: NCDB Analysis of Demographic Characteristics (displayed with percentages out of all patients)

| **Patient Variable** | **Total Patients, n (%)**  **(n = 40,164)** | **Initiation of PORT ≤ 6 Weeks, n (%)**  **(n = 15,263)** | **Initiation of PORT > 6 Weeks , n (%)**  **(n = 24,901)** | **OR**  **[95% CI]** |
| --- | --- | --- | --- | --- |
| **Age** | | | | |
| <50 | 5,258 (13.1%) | 2,122 (13.9%) | 3,136 (12.6%) | 1.00 [Ref] |
| 50-59 | 11,424 (28.4%) | 4,296 (28.1%) | 7,128 (28.6%) | 1.12 [1.05 - 1.20] |
| 60-69 | 13,397 (33.4%) | 5,063 (33.2%) | 8,334 (33.5%) | 1.11 [1.04 - 1.18] |
| >70 | 10,085 (25.1%) | 3,782 (24.8%) | 6,303 (25.3%) | 1.28 [1.05 - 1.20] |
| **Sex** | | | | |
| Male | 29,739 (74.0%) | 11,858 (77.7%) | 17,881 (71.8%) | 1.00 [Ref] |
| Female | 10,425 (26.0%) | 3,405 (22.3%) | 7,020 (28.2%) | 1.36 [1.30 - 1.43] |
| **Race** | | | | |
| White | 35,080 (87.3%) | 13,722 (89.9%) | 21,358 (85.8%) | 1.00 [Ref] |
| Black | 3,130 (7.8%) | 929 (6.1%) | 2,201 (8.8%) | 1.52 [1.40 - 1.64] |
| Asian/Pacific Islander | 1,193 (3.0%) | 361 (2.4%) | 832 (3.3%) | 1.48 [1.30 - 1.67] |
| Other/Unknown | 761 (1.9%) | 251 (1.6%) | 510 (2.0%) | 1.30 [1.12 - 1.52] |
| **Ethnicity** | | | | |
| Non-Hispanic | 37,629 (93.7%) | 14,408 (94.4%) | 23,221 (93.3%) | 1.00 [Ref] |
| Hispanic | 1,862 (4.6%) | 600 (3.9%) | 1,262 (5.1%) | 1.30 [1.18 - 1.44] |
| Unknown | 673 (1.7%) | 255 (1.7%) | 418 (1.7%) | 1.01 [0.86 - 1.19] |
| **Insurance Status** | | | | |
| Private Insurance | 1,140 (2.8%) | 344 (2.3%) | 796 (3.2%) | 1.00 [Ref] |
| Not Insured | 17,418 (43.4%) | 7,529 (49.3%) | 9,889 (39.7%) | 1.76 [1.54 - 2.00] |
| Medicaid | 4,591 (11.4%) | 1,216 (8.0%) | 3,375 (13.6%) | 2.11 [1.96 - 2.27] |
| Medicare | 15,551 (38.7%) | 5,644 (37.0%) | 9,907 (39.8%) | 1.33 [1.27 - 1.39] |
| Other Government | 983 (2.4%) | 316 (2.1%) | 667 (2.7%) | 1.60 [1.40 - 1.84] |
| Unknown | 481 (1.2%) | 214 (1.4%) | 267 (1.1%) | 0.95 [0.79 - 1.14] |
| **^1^Area of Residence** | | | | |
| Metropolitan | 32,187 (83.0%) | 12,150 (82.5%) | 20,037 (83.3%) | 1.00 [Ref] |
| Urban | 5,899 (15.2%) | 2,317 (15.7%) | 3,582 (14.9%) | 0.93 [0.87 - 0.99] |
| Rural | 686 (1.8%) | 264 (1.8%) | 422 (1.8%) | 0.93 [0.78 - 1.10] |
| **^1^Educational Attainment** | | | | |
| Highest Quartile | 8,386 (24.7%) | 3,571 (27.8%) | 4,815 (22.8%) | 1.00 [Ref] |
| Second Highest Quartile | 9,818 (28.9%) | 3,782 (29.5%) | 6,036 (28.5%) | 1.11 [1.03 - 1.20] |
| Second Lowest Quartile | 9,057 (26.7%) | 3,252 (25.4%) | 5,805 (27.5%) | 1.27 [1.18 - 1.37] |
| Lowest Quartile | 6,711 (19.8%) | 2,221 (17.3%) | 4,490 (21.2%) | 1.54 [1.43 - 1.67] |
| **^1^Median Household Income** | | | | |
| Less than $40,227 | 6,225 (18.4%) | 2,088 (16.3%) | 4,137 (19.6%) | 1.00 [Ref] |
| $40,227 - $50,353 | 7,660 (22.6%) | 2,809 (21.9%) | 4,851 (23.0%) | 0.87 [0.81 - 0.93] |
| $50,354 - $63,332 | 7,750 (22.9%) | 2,963 (23.1%) | 4,787 (22.7%) | 0.81 [0.76 - 0.87] |
| $63,333+ | 12,260 (36.2%) | 4,941 (38.6%) | 7,319 (34.7%) | 0.74 [0.70 - 0.79] |
| **Distance to Hospital, Miles** | | | | |
| ≤10 | 19,724 (49.1%) | 7,686 (50.4%) | 12,038 (48.3%) | 1.00 [Ref] |
| 11-20 | 6,669 (16.6%) | 2,593 (17.0%) | 4,076 (16.4%) | 1.00 [0.94 - 1.06] |
| 21-50 | 7,640 (19.0%) | 2,810 (18.4%) | 4,830 (19.4%) | 1.09 [1.03 - 1.15] |
| 51-10 | 3,683 (9.2%) | 1,232 (8.1%) | 2,451 (9.8%) | 1.27 [1.17 - 1.36] |
| >100 | 2,448 (6.1%) | 942 (6.2%) | 1,506 (6.0%) | 1.02 [0.93 - 1.11] |

Abbreviations: CI, confidence interval; OR, odds ratio; PORT, postoperative radiation therapy; Ref, reference category.

^1^Certain columns may not sum to the total due to missing observations.

Supplement 3: NCDB Analysis of Clinical and Surgical Characteristics (displayed with percentages out of all patients)

| **Patient Variable** | **Total Patients, n (%)**  **(n = 40,164)** | **Initiation of PORT ≤ 6 Weeks, n (%)**  **(n = 15,263)** | **Initiation of PORT > 6 Weeks , n (%)**  **(n = 24,901)** | **OR**  **[95% CI]** |
| --- | --- | --- | --- | --- |
| **Cancer Primary Site** | | | | |
| Oropharynx | 15,099 (37.6%) | 7,198 (47.2%) | 7,901 (31.7%) | 1.00 [Ref] |
| Oral Cavity | 16,415 (40.9%) | 4,297 (28.2%) | 12,118 (48.7%) | 2.56 [2.45 - 2.69] |
| Hypopharynx | 896 (2.2%) | 304 (2.0%) | 592 (2.4%) | 1.77 [1.53 - 2.04] |
| Larynx | 7,754 (19.3%) | 3,464 (22.7%) | 4,290 (17.2%) | 1.12 [1.06 - 1.19] |
| **^1^AJCC Clinical Stage Group** | | | | |
| I | 2,719 (13.8%) | 1,354 (17.5%) | 1,365 (11.4%) | 1.00 [Ref] |
| II | 2,766 (14.0%) | 985 (12.7%) | 1,781 (14.8%) | 1.79 [1.61 - 1.99] |
| III | 3,677 (18.6%) | 1,457 (18.9%) | 2,220 (18.5%) | 1.51 [1.36 - 1.67] |
| IV | 10,468 (53.0%) | 3,886 (50.3%) | 6,582 (54.7%) | 1.68 [1.54 - 1.82] |
| Unknown | 126 (0.6%) | 48 (0.6%) | 78 (0.6%) | 1.61 [1.11 - 2.32] |
| **^1^AJCC Pathological Stage Group** | | | | |
| I | 1,126 (3.3%) | 485 (4.0%) | 641 (2.9%) | 1.00 [Ref] |
| II | 1,406 (4.0%) | 468 (3.9%) | 938 (4.2%) | 1.51 [1.29 - 1.78] |
| III | 3,044 (8.8%) | 1,083 (8.9%) | 1,961 (8.7%) | 1.37 [1.19 - 1.57] |
| IV | 29,015 (83.9%) | 10,084 (83.2%) | 18,931 (84.2%) | 1.42 [1.25 - 1.60] |
| **^1^Surgical Margin Status** | | | | |
| Negative | 26,698 (66.5%) | 8,873 (58.1%) | 17,825 (71.6%) | 1.00 [Ref] |
| Positive | 10,596 (26.4%) | 4,811 (31.5%) | 5,785 (23.2%) | 0.59 [0.57 - 0.62] |
| Unknown | 2,870 (7.1%) | 1,579 (10.3%) | 1,291 (5.2%) | 0.40 [0.37 - 0.44] |
| **Postoperative Length of Stay, Days** | | | | |
| 0-3 | 18,596 (46.3%) | 9,205 (60.3%) | 9,391 (37.7%) | 1.00 [Ref] |
| 4-7 | 7,012 (17.5%) | 2,273 (14.9%) | 4,739 (19.0%) | 1.78 [1.67 - 1.90] |
| 8-14 | 7,664 (19.1%) | 1,928 (12.6%) | 5,736 (23.0%) | 2.04 [1.92 - 2.16] |
| 15-21 | 1,186 (3.0%) | 155 (1.0%) | 1,031 (4.1%) | 6.51 [5.49 - 7.73] |
| >21 | 1,042 (2.6%) | 132 (0.9%) | 910 (3.7%) | 6.75 [5.61 - 8.12] |
| Unknown | 4,664 (11.6%) | 1,570 (10.3%) | 3,094 (12.4%) | 1.93 [1.80 - 2.06] |
| **Readmission Within 30 Days of Discharge** | | | | |
| None | 37,474 (93.3%) | 14,428 (94.5%) | 23,046 (92.6%) | 1.00 [Ref] |
| Unplanned | 1,078 (2.7%) | 278 (1.8%) | 800 (3.2%) | 1.80 [1.56 – 2.06] |
| Planned | 726 (1.8%) | 269 (1.8%) | 457 (1.8%) | 1.06 [0.91 - 1.23] |
| Unknown | 886 (2.2%) | 288 (1.9%) | 598 (2.4%) | 1.30 [1.12 - 1.49] |
| **^1^Radiation Modality** | | | | |
| External Beam | 2,005 (5.1%) | 1,007 (6.7%) | 998 (4.1%) | 1.00 [Ref] |
| Conformal or 3D Therapy | 11,774 (29.7%) | 4,767 (31.6%) | 7,007 (28.5%) | 0.67 [0.61 – 0.74] |
| IMRT | 25,843 (65.2%) | 9,296 (61.7%) | 16,547 (67.4%) | 1.21 [1.15 - 1.26] |
| **Concurrent Chemoradiation** | | | | |
| No | 30,062 (74.8%) | 10,946 (71.7%) | 19,116 (76.8%) | 1.00 [Ref] |
| Yes | 10,102 (25.2%) | 4,317 (28.3%) | 5,785 (23.2%) | 0.76 [0.73 - 0.80] |
| **Charlson-Deyo Comorbidy Score** | | | | |
| 0 | 29,888 (74.4%) | 11,641 (76.3%) | 18,247 (73.3%) | 1.00 [Ref] |
| 1 | 6,765 (16.8%) | 2,390 (15.7%) | 4,375 (17.6%) | 1.16 [1.10 - 1.23] |
| ≥2 | 3,511 (8.7%) | 1,232 (8.1%) | 2,279 (9.2%) | 1.18 [1.09 - 1.27] |

Abbreviations: PORT, postoperative radiation therapy; OR, odds ratio; CI, confidence interval, Ref, reference category, AJCC, American Joint Committee on Cancer; 3D, 3-dimensional; IMRT, intensity-modulated radiation therapy.

^1^Certain columns may not sum to the total due to missing observations.

Supplement 4: NCDB Analysis of Hospital Characteristics (displayed with percentages out of all patients)

| **Patient Variable** | **Total Patients, n (%)**  **(n = 40,164)** | **Initiation of PORT ≤ 6 Weeks, n (%)**  **(n = 15,263)** | **Initiation of PORT > 6 Weeks , n (%)**  **(n = 24,901)** | **OR**  **[95% CI]** |
| --- | --- | --- | --- | --- |
| **^1^Treatment Facility Type** | | | | |
| Community Cancer Program | 1,909 (4.9%) | 797 (5.4%) | 1,112 (4.6%) | 1.00 [Ref] |
| Comprehensive Community Cancer Program | 10,436 (26.7%) | 4,524 (30.5%) | 5,912 (24.4%) | 0.93 [0.84 - 1.03] |
| Academic/Research Program | 20,557 (52.6%) | 6,905 (46.6%) | 13,652 (56.3%) | 1.41 [1.28 - 1.55] |
| Integrated Network Cancer Program | 6,165 (15.8%) | 2,606 (17.6%) | 3,559 (14.7%) | 0.97 [0.88 - 1.08] |
| **Number of Facilities Involved In Treatment** | | | | |
| All treatment at 1 CoC Facility | 30,255 (75.3%) | 11,823 (77.5%) | 18,432 (74.0%) | 1.00 [Ref] |
| Treatment at >1 CoC Facility | 9,909 (24.7%) | 3,440 (22.5%) | 6,469 (26.0%) | 1.20 [1.15 - 1.26] |
| **Surgery and Radiation at Same Facility** | | | | |
| Yes | 20,312 (50.6%) | 7,024 (46.0%) | 13,288 (53.4%) | 1.00 [Ref] |
| No | 19,852 (49.4%) | 8,239 (54.0%) | 11,613 (46.6%) | 1.34 [1.28 - 1.39] |
| **^1^Region of Care** | | | | |
| Northeast | 8,070 (20.7%) | 2,702 (18.2%) | 5,368 (22.1%) | 1.00 [Ref] |
| Midwest | 11,014 (28.2%) | 4,604 (31.0%) | 6,410 (26.4%) | 0.70 [0.66 - 0.74] |
| South | 14,025 (35.9%) | 5,296 (35.7%) | 8,729 (36.0%) | 0.83 [0.78 - 0.87] |
| West | 5,958 (15.3%) | 2,230 (15.0%) | 3,728 (15.4%) | 0.84 [0.78 - 0.90] |

Abbreviations: PORT, postoperative radiation therapy; OR, odds ratio; CI, confidence interval, Ref, reference category; CoC, Commission on Cancer.

^1^Certain columns may not sum to the total due to missing observations.

Supplement 5: Risk Factors for Prolonged Length of Stay (displayed with percentages out of all patients)

|  | **Postoperative Length of Stay (days)** | | | |  |  |
| --- | --- | --- | --- | --- | --- | --- |
| **Patient Variable** | 0-3 (n = 18,596) | 4-7 (n = 7,012) | 8-14 (n = 7,664) | 15-21 (n = 1,186) | >21 (n = 1,042) | **P-Value** |
| **Age**, n (%) | | | | |  |  |
| <50 | 2,392 (12.9%) | 1,006 (14.3%) | 1,016 (13.3%) | 137 (11.6%) | 106 (10.2%) | <0.001^1^ |
| 50-59 | 5,381 (28.9%) | 2,018 (28.8%) | 2,091 (27.3%) | 336 (28.3%) | 321 (30.8%) |  |
| 60-69 | 6,062 (32.6%) | 2,391 (34.1%) | 2,567 (33.5%) | 396 (33.4%) | 340 (32.6%) |  |
| >70 | 4,761 (25.6%) | 1,597 (22.8%) | 1,990 (26.0%) | 317 (26.7%) | 275 (26.4%) |  |
| **Sex**, n (%) | | | | |  |  |
| Male | 14,291 (76.8%) | 5,069 (72.3%) | 5295 (69.1%) | 817 (68.9%) | 739 (70.9%) | <0.001^1^ |
| Female | 4,305 (23.2%) | 1,943 (27.7%) | 2369 (30.9%) | 369 (31.1%) | 303 (29.1%) |  |
| **Race**, n (%) | | | | |  |  |
| White | 16,856 (90.6%) | 6,117 (87.2%) | 6,243 (81.5%) | 931 (78.5%) | 826 (79.3%) | <0.001^1^ |
| Black | 1,072 (5.8%) | 523 (7.5%) | 878 (11.5%) | 176 (14.8%) | 142 (13.6%) |  |
| Asian/Pacific Islander | 372 (2.0%) | 228 (3.3%) | 367 (4.8%) | 62 (5.2%) | 43 (4.1%) |  |
| Other/Unknown | 296 (1.6%) | 144 (2.1%) | 176 (2.3%) | 17 (1.4%) | 31 (3.0%) |  |
| **Ethnicity**, n (%) | | | | |  |  |
| Non-Hispanic | 17,538 (94.3%) | 6,566 (93.6%) | 7,095 (92.6%) | 1,106 (93.3%) | 957 (91.8%) | <0.001^1^ |
| Hispanic | 737 (4.0%) | 348 (5.0%) | 464 (6.1%) | 63 (5.3%) | 71 (6.8%) |  |
| Unknown | 321 (1.7%) | 98 (1.4%) | 105 (1.4%) | 17 (1.4%) | 14 (1.3%) |  |
| **Insurance Status**, n (%) | | | | |  |  |
| Not Insured | 383 (2.1%) | 227 (3.2%) | 323 (4.2%) | 46 (3.9%) | 46 (4.4%) | <0.001^1^ |
| Private Insurance | 9,049 (48.7%) | 3,123 (44.5%) | 2,700 (35.2%) | 334 (28.2%) | 259 (24.9%) |  |
| Medicaid | 1,424 (7.7%) | 875 (12.5%) | 1,305 (17.0%) | 239 (20.2%) | 255 (24.5%) |  |
| Medicare | 7,126 (38.3%) | 2,545 (36.3%) | 3,118 (40.7%) | 518 (43.7%) | 450 (43.2%) |  |
| Other Government | 398 (2.1%) | 152 (2.2%) | 146 (1.9%) | 33 (2.8%) | 21 (2.0%) |  |
| Insurance Status Unknown | 216 (1.2%) | 90 (1.3%) | 72 (0.9%) | 16 (1.3%) | 11 (1.1%) |  |
| **Urban/rural**, n (%) | | | | |  |  |
| Metropolitan | 15,006 (83.5%) | 5,525 (82.2%) | 6,115 (83.1%) | 974 (85.5%) | 853 (83.5%) | 0.01^1^ |
| Urban | 2,679 (14.9%) | 1,063 (15.8%) | 1,101 (15.0%) | 149 (13.1%) | 151 (14.8%) |  |
| Rural | 289 (1.6%) | 137 (2.0%) | 139 (1.9%) | 16 (1.4%) | 17 (1.7%) |  |
| **Education**, n (%) | | | | |  |  |
| Lowest Quartile | 2,707 (17.3%) | 1,131 (19.2%) | 1,530 (23.5%) | 279 (27.3%) | 250 (28.9%) | <0.001^1^ |
| Second Lowest Quartile | 4,010 (25.6%) | 1,595 (27.1%) | 1,807 (27.7%) | 295 (28.8%) | 216 (25.0%) |  |
| Second Highest Quartile | 4,661 (29.8%) | 1,671 (28.3%) | 1,795 (27.5%) | 267 (26.1%) | 250 (28.9%) |  |
| Highest Quartile | 4,260 (27.2%) | 1,499 (25.4%) | 1,391 (21.3%) | 182 (17.8%) | 149 (17.2%) |  |
| **Median Household Income**, n (%) | | | | |  |  |
| Less than $40,227 | 2,483 (15.9%) | 1,067 (18.1%) | 1,432 (22.0%) | 252 (24.7%) | 221 (25.7%) | <0.001^1^ |
| $40,227 - $50,353 | 3,380 (21.7%) | 1,358 (23.1%) | 1,500 (23.1%) | 220 (21.6%) | 181 (21.0%) |  |
| $50,354 - $63,332 | 3,564 (22.8%) | 1,336 (22.7%) | 1,479 (22.7%) | 241 (23.6%) | 203 (23.6%) |  |
| $63,333 + | 6,176 (39.6%) | 2,126 (36.1%) | 2,096 (32.2%) | 307 (30.1%) | 255 (29.7%) |  |
| **Great Circle Distance**, n (%) | | | | | | |
| ≤10 | 9,843 (52.9%) | 2912 (41.5%) | 3367 (43.9%) | 569 (48.0%) | 511 (49.0%) | <0.001^1^ |
| 11-20 | 3,251 (17.5%) | 1049 (15.0%) | 1158 (15.1%) | 190 (16.0%) | 153 (14.7%) |  |
| 21-50 | 3,327 (17.9%) | 1472 (21.0%) | 1585 (20.7%) | 221 (18.6%) | 198 (19.0%) |  |
| 51-10 | 1,386 (7.5%) | 896 (12.8%) | 928 (12.1%) | 124 (10.5%) | 120 (11.5%) |  |
| >100 | 7,89 (4.2%) | 683 (9.7%) | 626 (8.2%) | 82 (6.9%) | 60 (5.8%) |  |
| **Charlson-Deyo Score**, n (%) | | | | | | |
| 0 | 14,406 (77.5%) | 5,108 (72.8%) | 5,207 (67.9%) | 767 (64.7%) | 666 (63.9%) | <0.001^1^ |
| 1 | 2,816 (15.1%) | 1,260 (18.0%) | 1,589 (20.7%) | 263 (22.2%) | 217 (20.8%) |  |
| ≥2 | 1,374 (7.4%) | 644 (9.2%) | 868 (11.3%) | 156 (13.2%) | 159 (15.3%) |  |
| **Primary Site**, n (%) | | | | | | |
| Oral Cavity | 4,653 (25.0%) | 3,611 (51.5%) | 4933 (64.4%) | 816 (68.8%) | 630 (60.5%) | <0.001^1^ |
| Oropharynx | 9,981 (53.7%) | 2,099 (29.9%) | 908 (11.8%) | 133 (11.2%) | 148 (14.2%) |  |
| Hypopharynx | 253 (1.4%) | 168 (2.4%) | 270 (3.5%) | 41 (3.5%) | 52 (5.0%) |  |
| Larynx | 3,709 (19.9%) | 1,134 (16.2%) | 1,553 (20.3%) | 196 (16.5%) | 212 (20.3%) |  |
| **AJCC Clinical Stage Group**, n (%) | | | | | | |
| 0 | 152 (1.7%) | 12 (0.3%) | 7 (0.2%) | 1 (0.2%) | 1 (0.2%) | < 0.001^1^ |
| 1 | 1,963 (21.3%) | 237 (6.5%) | 168 (4.5%) | 18 (3.2%) | 20 (3.9%) |  |
| 2 | 1,307 (14.2%) | 553 (15.2%) | 500 (13.3%) | 64 (11.3%) | 57 (11.2%) |  |
| 3 | 1,692 (18.4%) | 752 (20.6%) | 651 (17.4%) | 88 (15.6%) | 72 (14.1%) |  |
| 4 | 4,041 (43.9%) | 2,071 (56.8%) | 2,405 (64.1%) | 387 (68.6%) | 358 (70.1%) |  |
| Unknown | 55 (0.6%) | 20 (0.5%) | 20 (0.5%) | 6 (1.1%) | 3 (0.6%) |  |
| **AJCC Pathological Stage Group**, n (%) | | | | | | |
| 0 | 93 (0.6%) | 2 (0.0%) | 2 (0.0%) | 1 (0.1%) | 0 (0.0%) | < 0.001^1^ |
| 1 | 753 (5.3%) | 139 (2.0%) | 89 (1.2%) | 7 (0.6%) | 15 (1.5%) |  |
| 2 | 582 (4.1%) | 315 (4.6%) | 273 (3.6%) | 39 (3.4%) | 30 (3.0%) |  |
| 3 | 1291 (9.0%) | 649 (9.5%) | 639 (8.5%) | 83 (7.1%) | 60 (5.9%) |  |
| 4 | 11,614 (81.0%) | 5,697 (83.8%) | 6,527 (86.7%) | 1,032 (88.8%) | 907 (89.6%) |  |
| **Surgical margin status**, n (%) | | | | | | |
| Negative | 10,154 (54.6%) | 5,761 (82.2%) | 6,279 (81.9%) | 927 (78.2%) | 771 (74.0%) | < 0.001^1^ |
| Positive | 6,251 (33.6%) | 1,145 (16.3%) | 1,300 (17.0%) | 245 (20.7%) | 253 (24.3%) |  |
| Unknown | 2,191 (11.8%) | 106 (1.5%) | 85 (1.1%) | 14 (1.2%) | 18 (1.7%) |  |
| **Readmission within 30 Days of Discharge**, n (%) | | | | | | |
| None | 17,683 (95.1%) | 6,514 (92.9%) | 7,137 (93.1%) | 1,080 (91.1%) | 950 (91.2%) | < 0.001^1^ |
| Unplanned | 306 (1.6%) | 291 (4.2%) | 326 (4.3%) | 77 (6.5%) | 61 (5.9%) |  |
| Planned | 362 (1.9%) | 146 (2.1%) | 149 (1.9%) | 24 (2.0%) | 21 (2.0%) |  |
| Unknown | 245 (1.3%) | 61 (0.9%) | 52 (0.7%) | 5 (0.4%) | 10 (1.0%) |  |
| **Radiation modality**, n (%) | | | | | | |
| Conformal or 3D therapy | 1,302 (7.1%) | 193 (2.8%) | 185 (2.4%) | 35 (3.0%) | 29 (2.8%) | < 0.001^1^ |
| External beam | 5,679 (31.0%) | 2,032 (29.3%) | 2,133 (28.1%) | 333 (28.4%) | 265 (25.8%) |  |
| IMRT | 11,314 (61.8%) | 4,715 (67.9%) | 5,269 (69.4%) | 805 (68.6%) | 734 (71.4%) |  |
| **Concurrent Chemoradiation**, n (%) | | | | | | |
| No | 13,834 (74.4%) | 5,320 (75.9%) | 5,760 (75.2%) | 899 (75.8%) | 788 (75.6%) | 0.14^1^ |
| Yes | 4,762 (25.6%) | 1,692 (24.1%) | 1,904 (24.8%) | 287 (24.2%) | 254 (24.4%) |  |

^1^Chi-Square p-value.

Abbreviations: AJCC, American Joint Committee on Cancer; 3D, 3-dimensional; IMRT, intensity-modulated radiation therapy.

^1^Certain columns may not sum to the total due to missing observations.

Supplement 6: Risk Factors for Hospital Readmission (displayed with percentages out of all patients)

|  | **Readmission Within 30 Days of Discharge** | | | |  |
| --- | --- | --- | --- | --- | --- |
| **Patient Variable** | **None (n = 37,474)** | **Unplanned (n = 1,078)** | **Planned (n = 726)** | **Unknown (n = 886)** | **P-Value** |
| **Age**, n (%) | | | | | |
| <50 | 4,870 (13.0%) | 164 (15.2%) | 106 (14.6%) | 118 (13.3%) | 0.14^1^ |
| 50-59 | 10,656 (28.4%) | 320 (29.7%) | 210 (28.9%) | 238 (26.9%) |  |
| 60-69 | 12,494 (33.3%) | 343 (31.8%) | 253 (34.8%) | 307 (34.7%) |  |
| >70 | 9,454 (25.2%) | 251 (23.3%) | 157 (21.6%) | 223 (25.2%) |  |
| **Sex**, n (%) | | | | | |
| Male | 27,766 (74.1%) | 789 (73.2%) | 533 (73.4%) | 651 (73.5%) | 0.86^1^ |
| Female | 9,708 (25.9%) | 289 (26.8%) | 193 (26.6%) | 235 (26.5%) |  |
| **Race**, n (%) | | | | | |
| White | 32,794 (87.5%) | 911 (84.5%) | 612 (84.3%) | 763 (86.1%) | <0.001^1^ |
| Black | 2878 (7.7%) | 118 (10.9%) | 65 (9.0%) | 69 (7.8%) |  |
| Asian/Pacific Islander | 1,105 (2.9%) | 30 (2.8%) | 31 (4.3%) | 27 (3.0%) |  |
| Other/Unknown | 697 (1.9%) | 19 (1.8%) | 18 (2.5%) | 27 (3.0%) |  |
| **Ethnicity**, n (%) | | | | | |
| Non-Hispanic | 35,092 (93.6%) | 1,028 (95.4%) | 690 (95.0%) | 819 (92.4%) | 0.10^1^ |
| Hispanic | 1751 (4.7%) | 36 (3.3%) | 28 (3.9%) | 47 (5.3%) |  |
| Unknown | 631 (1.7%) | 14 (1.3%) | 8 (1.1%) | 20 (2.3%) |  |
| **Insurance Status**, n (%) | | | | | |
| Not Insured | 1,067 (2.8%) | 27 (2.5%) | 29 (4.0%) | 17 (1.9%) | <0.001^1^ |
| Private Insurance | 16,332 (43.6%) | 412 (38.2%) | 323 (44.5%) | 351 (39.6%) |  |
| Medicaid | 4,257 (11.4%) | 152 (14.1%) | 77 (10.6%) | 105 (11.9%) |  |
| Medicare | 14,499 (38.7%) | 453 (42.0%) | 259 (35.7%) | 340 (38.4%) |  |
| Other Government | 895 (2.4%) | 23 (2.1%) | 17 (2.3%) | 48 (5.4%) |  |
| Insurance Status Unknown | 424 (1.1%) | 11 (1.0%) | 21 (2.9%) | 25 (2.8%) |  |
| **Urban/rural**, n (%) | | | | | |
| Metropolitan | 30,038 (83.1%) | 880 (83.7%) | 607 (84.9%) | 662 (77.0%) | <0.001^1^ |
| Urban | 5,475 (15.1%) | 148 (14.1%) | 100 (14.0%) | 176 (20.5%) |  |
| Rural | 633 (1.8%) | 23 (2.2%) | 8 (1.1%) | 22 (2.6%) |  |
| **Education**, n (%) | | | | | |
| Lowest Quartile | 6,213 (19.6%) | 209 (23.0%) | 125 (20.3%) | 164 (22.0%) | 0.01^1^ |
| Second Lowest Quartile | 8,451 (26.7%) | 238 (26.2%) | 148 (24.1%) | 220 (29.5%) |  |
| Second Highest Quartile | 9,183 (29.0%) | 239 (26.3%) | 181 (29.4%) | 215 (28.8%) |  |
| Highest Quartile | 7,855 (24.8%) | 222 (24.4%) | 161 (26.2%) | 148 (19.8%) |  |
| **Median Household Income**, n (%) | | | | | |
| Less than $40,227 | 5,734 (18.1%) | 206 (22.7%) | 113 (18.4%) | 172 (23.1%) | 0.001^1^ |
| $40,227 - $50,353 | 7,181 (22.7%) | 183 (20.2%) | 130 (21.1%) | 166 (22.3%) |  |
| $50,354 - $63,332 | 7,264 (23.0%) | 195 (21.5%) | 134 (21.8%) | 157 (21.1%) |  |
| $63,333 + | 11,450 (36.2%) | 322 (35.5%) | 238 (38.7%) | 250 (33.6%) |  |
| **Great Circle Distance**, n (%) | | | | | |
| ≤10 | 18,298 (48.8%) | 519 (48.1%) | 362 (49.9%) | 545 (61.5%) | <0.001^1^ |
| 11-20 | 6,237 (16.6%) | 161 (14.9%) | 120 (16.5%) | 151 (17.0%) |  |
| 21-50 | 7,191 (19.2%) | 214 (19.9%) | 118 (16.3%) | 117 (13.2%) |  |
| 51-10 | 3,458 (9.2%) | 106 (9.8%) | 80 (11.0%) | 39 (4.4%) |  |
| >100 | 2,290 (6.1%) | 78 (7.2%) | 46 (6.3%) | 34 (3.8%) |  |
| **Charlson-Deyo Score**, n (%) | | | | | |
| 0 | 27,911 (74.5%) | 719 (66.7%) | 529 (72.9%) | 729 (82.3%) | <0.001^1^ |
| 1 | 6,302 (16.8%) | 220 (20.4%) | 129 (17.8%) | 114 (12.9%) |  |
| ≥2 | 3,261 (8.7%) | 139 (12.9%) | 68 (9.4%) | 43 (4.9%) |  |
| **Primary Site**, n (%) | | | | | |
| Oral Cavity | 15,227 (40.6%) | 533 (49.4%) | 294 (40.5%) | 361 (40.7%) | <0.001^1^ |
| Oropharynx | 14,154 (37.8%) | 323 (30.0%) | 279 (38.4%) | 343 (38.7%) |  |
| Hypopharynx | 812 (2.2%) | 38 (3.5%) | 17 (2.3%) | 29 (3.3%) |  |
| Larynx | 7,281 (19.4%) | 184 (17.1%) | 136 (18.7%) | 153 (17.3%) |  |
| **AJCC Clinical Stage Group**, n (%) | | | | | |
| 1 | 2,594 (13.9%) | 32 (6.3%) | 48 (12.6%) | 45 (12.1%) | <0.001^1^ |
| 2 | 2,610 (14.0%) | 63 (12.4%) | 51 (13.4%) | 42 (11.3%) |  |
| 3 | 3,444 (18.4%) | 99 (19.5%) | 62 (16.2%) | 72 (19.3%) |  |
| 4 | 9,733 (52.1%) | 308 (60.7%) | 216 (56.5%) | 211 (56.6%) |  |
| Unknown | 117 (0.6%) | 3 (0.6%) | 4 (1.0%) | 2 (0.5%) |  |
| **AJCC Pathological Stage Group**, n (%) | | | | | |
| 1 | 1,072 (3.3%) | 17 (1.7%) | 18 (2.8%) | 19 (2.4%) | 0.02^1^ |
| 2 | 1,335 (4.1%) | 30 (3.0%) | 20 (3.1%) | 21 (2.7%) |  |
| 3 | 2,819 (8.7%) | 84 (8.4%) | 62 (9.6%) | 79 (10.2%) |  |
| 4 | 26,946 (83.5%) | 866 (86.8%) | 547 (84.3%) | 656 (84.5%) |  |
| **Surgical margin status**, n (%) | | | | | |
| Negative | 24,882 (66.4%) | 790 (73.3%) | 506 (69.7%) | 520 (58.7%) | <0.001^1^ |
| Positive | 9,904 (26.4%) | 261 (24.2%) | 184 (25.3%) | 247 (27.9%) |  |
| Unknown | 2,688 (7.2%) | 27 (2.5%) | 36 (5.0%) | 119 (13.4%) |  |
| **Postoperative Length of Stay, days**, n (%) | | | | | |
| 0-3 | 17,683 (53.0%) | 306 (28.8%) | 362 (51.6%) | 245 (65.7%) | <0.001^1^ |
| 4-7 | 6,514 (19.5%) | 291 (27.4%) | 146 (20.8%) | 61 (16.4%) |  |
| 8-14 | 7,137 (21.4%) | 326 (30.7%) | 149 (21.2%) | 52 (13.9%) |  |
| 15-21 | 1,080 (3.2%) | 77 (7.3%) | 24 (3.4%) | 5 (1.3%) |  |
| >21 | 950 (2.8%) | 61 (5.7%) | 21 (3.0%) | 10 (2.7%) |  |
| **Radiation modality**, n (%) | | | | | |
| Conformal or 3D therapy | 1,902 (5.1%) | 40 (3.7%) | 24 (3.3%) | 39 (4.4%) | 0.003^1^ |
| External beam | 11,044 (29.9%) | 291 (27.1%) | 200 (27.9%) | 239 (27.3%) |  |
| IMRT | 24,007 (65.0%) | 743 (69.2%) | 494 (68.8%) | 599 (68.3%) |  |
| **Concurrent Chemoradiation**, n (%) | | | | | |
| No | 28,048 (74.8%) | 825 (76.5%) | 534 (73.6%) | 655 (73.9%) | 0.45^1^ |
| Yes | 9,426 (25.2%) | 253 (23.5%) | 192 (26.4%) | 231 (26.1%) |  |

^1^Chi-Square p-value.

Abbreviations: AJCC, American Joint Committee on Cancer; 3D, 3-dimensional; IMRT, intensity-modulated radiation therapy.

Supplement 7: TriNetX Analysis of Demographic Characteristics (displayed with percentages out of all patients)

| **Patient Variable** | **Total Patients, n (%)**  **(n = 3,165)** | **Initiation of PORT**  **≤ 6 Weeks, n (%)**  **(n = 1,142)** | **Initiation of PORT**  **> 6 Weeks, n (%)**  **(n = 2,023)** | **OR**  **[95% CI]** |
| --- | --- | --- | --- | --- |
| **Age** | | | | |
| < 65 years | 1,570 (49.6%) | 564 (49.4%) | 1,006 (49.7%) | 1 [Ref] |
| ≥ 65 years | 1,595 (50.4%) | 578 (50.6%) | 1,017 (50.3%) | 0.99 [0.85 - 1.01] |
| **Sex** | | | | |
| Male | 2,331 (73.6%) | 874 (76.5%) | 1,457 (72.0%) | 1 [Ref] |
| Female | 834 (26.4%) | 268 (23.5%) | 566 (28.0%) | 1.27 [1.08 - 1.49] |
| **Race** | | | | |
| White | 2,554 (80.7%) | 937 (82.1%) | 1,617 (79.9%) | 1 [Ref] |
| Black | 387 (12.2%) | 131 (11.5%) | 256 (12.7%) | 1.14 [0.91 - 1.43] |
| Asian | 57 (1.8%) | 21 (1.8%) | 36 (1.8%) | 0.99 [0.58 - 1.72] |
| Other/Unknown | 167 (5.3%) | 53 (4.6%) | 114 (5.6%) | 1.25 [0.89 - 1.75] |
| **Ethnicity** | | | | |
| Not Hispanic or Latino | 2,622 (82.8%) | 969 (84.9%) | 1,653 (81.7%) | 1 [Ref] |
| Hispanic or Latino | 179 (5.7%) | 62 (5.4%) | 117 (5.8%) | 1.11 [0.81 - 1.52] |
| Other/Unknown | 364 (11.5%) | 111 (9.7%) | 253 (12.5%) | 1.33 [1.05 - 1.69] |
| **Marital Status** | | | | |
| Married | 966 (30.6%) | 422 (37.0%) | 544 (26.9%) | 1 [Ref] |
| Never Married | 418 (13.2%) | 142 (12.4%) | 276 (13.6%) | 1.51 [1.19 - 1.92] |
| Divorced | 194 (6.1%) | 69 (6.1%) | 125 (6.2%) | 1.41 [1.02 - 1.92] |
| Widowed | 133 (4.2%) | 46 (4.0%) | 87 (4.3%) | 1.47 [1.01 - 2.13] |
| Other/Unknown | 1,454 (45.9%) | 463 (40.5%) | 991 (49.0%) | 1.67 [1.41 - 1.96] |
| **Region of Care** |  |  |  |  |
| East | 2,041 (64.5%) | 731 (64.0%) | 1,310 (64.8%) | 1 [Ref] |
| West | 1,112 (35.1%) | 408 (35.7%) | 704 (34.8%) | 0.96 [0.83 - 1.12] |
| Unknown | 12 (0.4%) | 3 (0.3%) | 9 (0.4%) | 1.67 [0.45 - 6.25] |

Abbreviations: PORT, postoperative radiation therapy; OR, odds ratio; CI, confidence interval, Ref, reference category.

Supplement 8: TriNetX Analysis of Clinical and Surgical Characteristics (displayed with percentages out of all patients)

| **Patient Variable** | **Total Patients, n (%)**  **(n = 3,165)** | **Initiation of PORT**  **≤ 6 Weeks, n (%)**  **(n = 1,142)** | **Initiation of PORT**  **> 6 Weeks, n (%)**  **(n = 2,023)** | **OR**  **[95% CI]** |
| --- | --- | --- | --- | --- |
| **Cancer Site** | | | | |
| Oropharynx | 1,116 (35.2%) | 485 (42.5%) | 631 (31.2%) | 1 [Ref] |
| Oral Cavity | 1,376 (43.5%) | 412 (36.1%) | 964 (47.7%) | 1.79 [1.52 – 2.12] |
| Hypopharynx | 98 (3.1%) | 46 (4.0%) | 52 (2.6%) | 0.87 [0.57 - 1.31] |
| Larynx | 575 (18.2%) | 199 (17.4%) | 376 (18.5%) | 1.45 [1.18 – 1.79] |
| **Concurrent Chemoradiation** | | | | |
| No | 1,947 (61.5%) | 660 (57.8%) | 1,287 (63.6%) | 1 [Ref] |
| Yes | 1,218 (38.5%) | 482 (42.2%) | 736 (36.4%) | 0.78 [0.68 - 0.91] |
| **Neck Dissection** | | | | |
| No | 1,387 (43.8%) | 579 (50.7%) | 808 (39.9%) | 1 [Ref] |
| Yes | 1,778 (56.2%) | 563 (49.3%) | 1,215 (60.1%) | 1.55 [1.34 - 1.79] |
| **Free Skin Flap** | | | | |
| No | 2,755 (87.0%) | 997 (87.3%) | 1,758 (86.9%) | 1 [Ref] |
| Yes | 410 (13.0%) | 145 (12.7%) | 265 (13.1%) | 1.04 [0.83 - 1.29] |
| **Free Osteocutaneous Flap** | | | | |
| No | 2,839 (89.7%) | 1,057 (7.4%) | 1,782 (88.1%) | 1 [Ref] |
| Yes | 326 (10.3%) | 85 (92.6%) | 241 (11.9%) | 1.68 [1.30 - 2.18] |
| **Free Muscle or Myocutaneous Flap** | | | | |
| No | 3,008 (95.0%) | 1,113 (97.5%) | 1,895 (93.7%) | 1 [Ref] |
| Yes | 157 (5.0%) | 29 (2.5%) | 128 (6.3%) | 2.59 [1.72 - 3.91] |
| **Free Fascial Flap** | | | | |
| No | 3,081 (97.3%) | 1,125 (98.5%) | 1,956 (96.7%) | 1 [Ref] |
| Yes | 84 (2.7%) | 17 (1.5%) | 67 (3.3%) | 2.27 [1.32 - 3.88] |
| **Laryngectomy** | | | | |
| No | 2,839 (89.7%) | 1,067 (93.4%) | 1,772 (87.6%) | 1 [Ref] |
| Yes | 326 (10.3%) | 75 (6.6%) | 251 (12.4%) | 2.01 [1.54 - 2.64] |
| **Gastrostomy Status** | | | | |
| No | 2,030 (64.1%) | 776 (68.0%) | 1,254 (62.0%) | 1 [Ref] |
| Yes | 1,135 (35.9%) | 366 (32.0%) | 769 (38.0%) | 1.30 [1.11 - 1.51] |
| **Tracheostomy Status** | | | | |
| No | 2,234 (70.6%) | 880 (77.1%) | 1,354 (66.9%) | 1 [Ref] |
| Yes | 931 (29.4%) | 262 (22.9%) | 669 (33.1%) | 1.67 [1.41 - 1.96] |

Abbreviations: PORT, postoperative radiation therapy; OR, odds ratio; CI, confidence interval, Ref, reference category.
